# Cloxyquin activates hTRESK by allosteric modulation of the selectivity filter

**DOI:** 10.1038/s42003-023-05114-4

**Published:** 2023-07-18

**Authors:** Julian Alexander Schreiber, Anastasia Derksen, Gunnar Goerges, Sven Schütte, Jasmin Sörgel, Aytug K. Kiper, Nathalie Strutz-Seebohm, Tobias Ruck, Sven G. Meuth, Niels Decher, Guiscard Seebohm

**Affiliations:** 1 Institute for Genetics of Heart Diseases (IfGH), Department of Cardiovascular Medicine, University Hospital Münster, Robert-Koch-Str. 45, Münster, Germany; 2 Westfälische Wilhelms-Universität Münster, Institut für Pharmazeutische und Medizinische Chemie, Corrensstr. 48, Münster, Germany; 3 Institute of Physiology and Pathophysiology, Vegetative Physiology, Philipps-University Marburg, Marburg, Germany; 4 Department of Neurology, Medical Faculty, Heinrich-Heine University, Düsseldorf, Germany; 5 Westfälische Wilhelms-Universität Münster, GRK 2515, Chemical biology of ion channels (Chembion), Münster, Germany

**Keywords:** Potassium channels, Drug development

## Abstract

The TWIK-related spinal cord K^+^ channel (TRESK, K_2P_18.1) is a K_2P_ channel contributing to the maintenance of membrane potentials in various cells. Recently, physiological TRESK function was identified as a key player in T-cell differentiation rendering the channel a new pharmacological target for treatment of autoimmune diseases. The channel activator cloxyquin represents a promising lead compound for the development of a new class of immunomodulators. Identification of cloxyquin binding site and characterization of the molecular activation mechanism can foster the future drug development. Here, we identify the cloxyquin binding site at the M2/M4 interface by mutational scan and analyze the molecular mechanism of action by protein modeling as well as in silico and in vitro electrophysiology using different permeating ion species (K^+^ / Rb^+^). In combination with kinetic analyses of channel inactivation, our results suggest that cloxyquin allosterically stabilizes the inner selectivity filter facilitating the conduction process subsequently activating hTRESK.

## Abstract (web summary)

Here, the binding site of the drug cloxyquin to the TWIK-related spinal cord K+ channel (TRESK) channel is determined and it is reported that Cloxyquin activates the ion channel by allosteric stabilization of the inner selectivity filter facilitating the ion conduction process.

## Introduction

Two-pore domain potassium (K_2P_) channels are expressed in a plethora of cells conducting K^+^ from the cytosol to the extracellular space and thereby lowering the membrane potential^1–3^. 15 mammalian genes encoding different subunits are known forming homo- and heterodimeric ion channels^4–6^. Each subunit possesses four transmembrane helices (M1-M4), two pore helices (P1, P2) and pore-forming loops (SF1, SF2) as well as two extracellular helices (C1, C2), encompassing the Cap structure typical for K_2P_ channels^7^. Like voltage-gated K^+^ channels (K_v_), dimeric K_2P_ channels assemble with a tetrameric selectivity filter (SF), which is formed by a pair of two inner pore loops (SF1, SF2) present in each monomer^7^. On the other hand, K_2P_ channels are not primary gated by voltage and adopt conductive states over a broad range of physiological voltages^8^. The SF exerts a dominant function in channel gating, which is mechanistically reminiscent of C-type inactivation from K_v_ channels^9–16^.

In contrast to other K_2P_ channels, the TWIK-related spinal cord K^+^ channel (TRESK, K_2P_18.1) is regulated by intracellular Ca^2+^ ions^17,18^. This regulation is controlled by calcineurin binding to an intracellular loop between M2 and M3, which is unique in K_2P_ channels^17,19^. Dephosphorylation at different positions in this loop leads to channel activation^18,20,21^. K_2P_18.1 expression is found in different regions of the nervous system like such as spinal cord, the cerebrum, the suprachiasmatic nucleus as well as the dorsal root and trigeminal ganglia^22–25^. Together with TREK-1/-2 (K_2P_2.1, K_2P_10.1), K_2P_18.1 lowers the membrane potential of these cells regulating neuronal activity^23–25^. Therefore, it is not surprising that dysfunctions are associated with disorders such as migraine, epilepsy, and neuropathic pain^18,26–28^. Very recently, K_2P_18.1 was identified as a master switch in regulatory T (T_reg_) cell proliferation and differentiation in the thymus^29^. Therefore, pharmacological activation of K_2P_18.1 could be a new strategy for immunomodulation in the treatment of autoimmune diseases like multiple sclerosis^29^.

The first promising lead compound is cloxyquin (5-chloro-8-hydroxyquinoline), which was identified as a K_2P_18.1 activator by Tl^+^ flux screening and whole cell patch clamp recordings^30^. Selectivity screenings proofed that cloxyquin specifically activates K_2P_18.1, while other K_2P_ channels were neither significantly inhibited nor activated^31^. Furthermore, channel stimulation by cloxyquin was identified as independent from indirect stimulation by Ca^2+^/calcineurin indicating a direct effect on the ion channel^31^. The direct stimulation was further supported by mutational studies at the murine K_2P_18.1 (mTRESK) channel. Replacement of the amino acids F156 and F364 by alanine resulted in a pronounced loss of cloxyquin activity, which might be induced by disruption of direct interactions with the channel. However, both mutations led to a strong gain of ion channel function, which could influence the effectiveness as well^31^. Although cloxyquin is highly selective over other K_2P_ channels, its moderate potency at human K_2P_18.1 (hTRESK) channels limits the applicability as a potential drug^30,31^. Using cloxyquin as a lead compound offers the opportunity to develop a new class of highly specific immunomodulators.

Here, we report the elucidation of the cloxyquin binding site and the molecular base for hTRESK channel activation by cloxyquin. Compound screening and mutational analyses identified crucial scaffold substitution pattern for activity as well as localization of the binding site. Furthermore, a molecular mechanism of action was assessed by homology modeling, molecular dynamic (MD) simulations and in silico electrophysiology. The results lead to the hypothesis, that cloxyquin binds to the M2/M4 helix interface leading to long range M4/M4 and M4/SF coupling subsequently stabilizing the SF, which is crucial for the overall conductance process^32^. This hypothesis is confirmed by Two-electrode voltage clamp (TEVC) recordings with different permeable ions (K^+^ / Rb^+^) as well as kinetic analysis of channel inactivation, which is effectively compensated by cloxyquin.

## Results

### A distinct substitution pattern is needed for activity

**Fig. 1:**
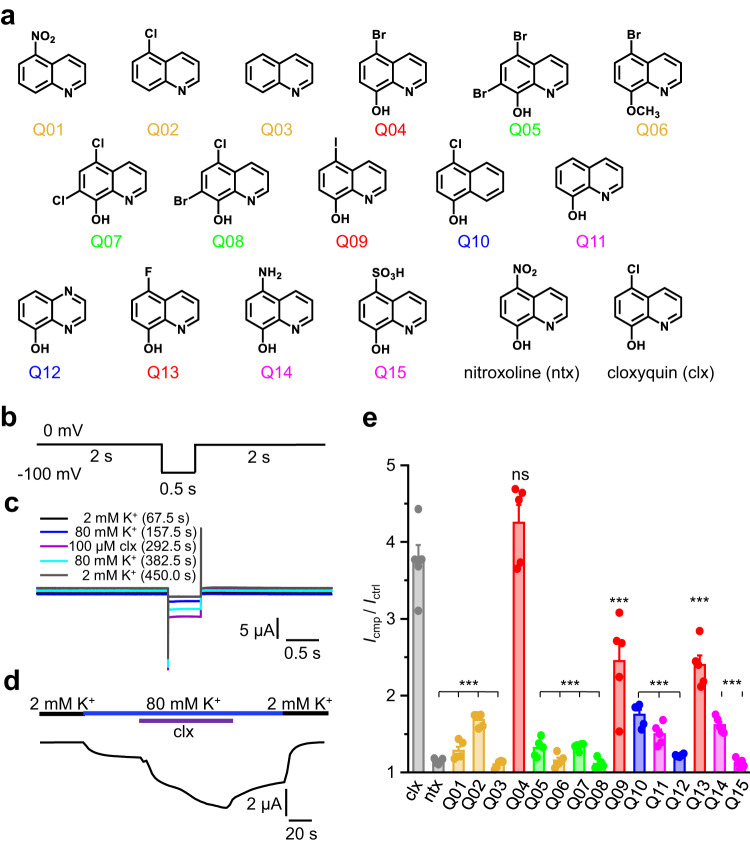
Structure activity screening of quinoline analogs Q01–Q15. **a** Structures of analyzed cloxyquin analogs Q01-Q15 compared to nitroxoline (ntx) and cloxyquin (clx). Colors indicate different substitution patterns corresponding to colors used in panel (**e**): Reference compounds (gray), quinolines without 8-OH-group (yellow), 5-halogenated 8-quinolinols (red), di-halogenated 8-quinolinols (green), quinoline analogous ring systems (blue) and 5-non-haloginated 8-quinolinols (magenta). **b** Pulse protocol for initial 100 µM screening. The protocol was repeated 100 times resulting in a total recording time of 450 s. **c** Overlay of repetitive pulse protocol application in presence of 2 mM K^+^ (67.5 s, black), 80 mM K^+^ (157.5 s, blue), 80 mM K^+^ and 100 µM cloxyquin (clx, 292.5 s, purple) followed by 80 mM K^+^ (382.5 s, cyan) and 2 mM K^+^ (450 s, black) washout. **d** Sample trace generated from repetitive pulse protocol application. The representative line graph was generated from 100 data points of a single oocyte and displays the change of current at -100 mV measured every 4.5 s. **e** Mean current potentiation ± SEM (*n* = 5 independent oocytes per compound) caused by application of 100 µM cloxyquin and nitroxoline compared to application of 100 µM Q01-Q15. Colors are defined in a. Current in presence of compound was normalized to the current in absence of the compound for each oocyte. Significance of mean differences to mean activation caused by 100 µM cloxyquin was tested by one-way ANOVA and post hoc mean comparison Tukey test and is indicated by ns for *p* > 0.05 and *** for *p* < 0.001.

To analyze the cloxyquin effect in detail, we examined the substitution pattern crucial for the agonistic effect. 15 derivatives (Q01-Q15) were screened for their capability to potentiate currents (*I*_cmp_ / *I*_ctrl_) in TEVC recordings using hTRESK wildtype (WT) expressing oocytes (Fig. 1a–d). All compounds were tested at a concentration of 100 µM in 80 mM K^+^ solution as previously described^31^. The examined current potentiation was compared to the effect of reference compounds cloxyquin and nitroxoline (Fig. 1e, Supplementary Tables 1 and 2)^29^. With exception of the 5-Br analog Q04, all tested derivatives are significantly less active. Interestingly, the 5-halogenated quinoline-8-ols Q13 (5-F), cloxyquin (5-Cl), Q04 (5-Br) and Q09 (5-I) are significantly more active than all other tested derivatives potentiating currents by at least factor 2.4 (Q13). While activity of Q09 is comparable to Q13, cloxyquin and Q04 potentiate the current even more rendering these two the most effective ones of all tested compounds. Contrary to halogenation in 5-position, incorporation of a second halogen atom in 7-position (Q05, Q07, Q08) almost eliminates the agonistic effect. Similar to these findings, the exchange of the halogen atom in 5-position by a nitro (nitroxoline, Q01), an amino (Q14) or a sulfo group (Q15) clearly reduces the agonistic effect underlining the importance of the halogen atom in 5-position. Comparison of current potentiation by cloxyquin to 5-chloroquinoline Q02 (1.68 ± 0.04) as well as to the fully unsubstituted quinoline Q03 (1.10 ± 0.02) leads to the conclusion that not only halogen substituents in 5-position but also the OH-group in 8-position is mandatory for activity. In line with this, the non-halogenated 8-quinolinol (Q11) shows significantly lower current potentiation (1.50 ± 0.06) compared to cloxyquin, which is similar to current potentiation of Q02. These results indicate that both substituents contribute synergistically to the agonistic effect, which is further supported by the low current potentiation caused by the 8-methoxy-derivative Q06. Furthermore, replacement of the quinoline ring system by a naphthalene (Q10) or quinoxaline (Q12) leads to reduced or equal current potentiation compared with the corresponding quinoline derivatives cloxyquin (for Q10) or Q11 (for Q12) indicating relevance of the ring system for the agonistic mechanism as well.

### Basic functional analysis of agonism and putative cloxyquin binding site prediction

**Fig. 2:**
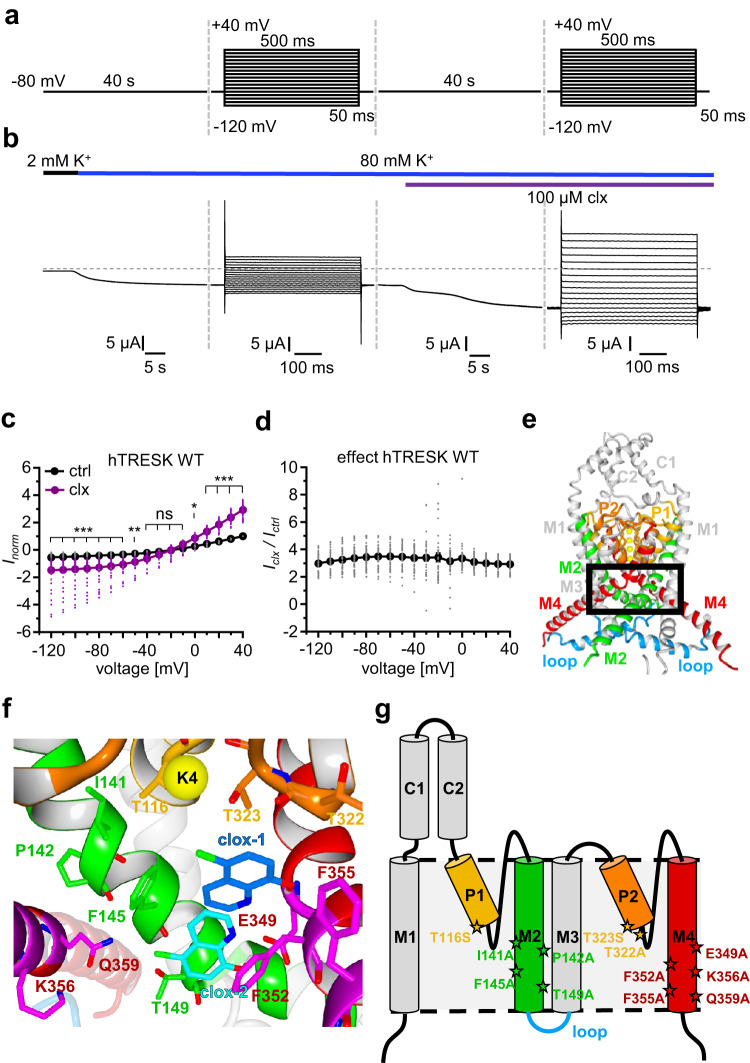
Voltage dependence of agonism and in silico hTRESK modeling. **a** IV pulse protocol. **b** Sample trace for hTRESK WT resulting from pulse protocol application in presence of 80 mM K^+^ and 100 µM cloxyquin. **c** Dot plot and mean ± SEM of normalized currents in absence (black) and presence (purple) of 100 µM cloxyquin calculated from *n* = 39 independent oocytes. Currents were normalized for each oocyte to the current in absence of cloxyquin at +40 mV. Significance of mean differences was analyzed by one-way ANOVA and post hoc mean comparison Tukey test. Significance levels are given for each voltage step and indicated by ns for *p* > 0.05, * for *p* < 0.05, ** for *p* < 0.01 and *** for *p* < 0.001. **d** Dot plot and corresponding mean ± SEM current potentiation caused by application of 100 µM cloxyquin at hTRESK WT calculated for each oocyte by division of current in presence of cloxyquin *I*_*clx*_ divided by current in absence of cloxyquin *I*_ctrl_ (*n* = 39). **e** Depiction of hTRESK model generated by dimerization of *AlphaFold* structure. Intracellular loop between M2 and M3 was replaced by flexible distance between residues P170 and P254. Zoom in area of panel (f) is highlighted by black box. **f** Depiction of potential cloxyquin binding sites clox-1/clox-2 as well as potential interacting residues. **g** Schematic depiction of hTRESK WT monomer and localization of generated mutants for binding site analyses.

Similar to K_v_ channels, TRESK channels feature an outward rectification depending on extracellular K^+^ concentration, which was recently linked to conformational stability of the SF^32,33^. To elucidate voltage dependency of cloxyquin agonism, the effect of 100 µM cloxyquin was examined at different voltages (Fig. 2a–d). In presence of 80 mM K^+^ 100 µM cloxyquin significantly increases inward as well as outward directed currents (*I*_norm_; Fig. 2c). Noteworthy, wash in of cloxyquin results in a biphasic current amplification characterized by a temporary steady state 10–20 s after compound application (Fig. 2b). Nevertheless, current potentiation (*I*_clx_ / *I*_ctrl_) is not altered at different voltages suggesting a voltage-independent agonism (Fig. 2d, Supplementary Table 3).

Based on the previous studies, it is plausible that the observed voltage-independent agonism can be allocated to direct interactions between cloxyquin and hTRESK^30,31,34^. Mutational analysis using mTRESK channel expressing oocytes identified F156 and F364 as potential interaction partners of cloxyquin^31^. However, neither structural information from crystal / cryo-EM structures nor additional interacting amino acids for the hTRESK channel are known. Therefore, we used protein sequence alignment and in silico modeling to identify potential homologous as well as further interacting amino acids at the hTRESK channel (Fig. 2e, Supplementary Figs. 1–2).

Protein sequence alignment of hTRESK identified F145 in the M2 and F352 in the M4 helix as orthologues of mTRESK F156 and F364 (Supplementary Fig. 1). Both phenylalanine side chains face to the inner cavity of the hTRESK model below the SF, which was previously also assumed for the mTRESK ortholouges^31^.

To identify potential binding sites in spatial proximity to hTRESK F145 and F352, we performed local *docking* at the inner cavity (Fig. 2f). *Docking* poses for cloxyquin cluster at two different positions, that were named clox-1 and clox-2. Furthermore, an additional binding site formed by M2 residues I141, P142, F145 and M4 residue K356 can be detected, that is also present in other K_2P_ channels forming a binding site for different modulators like BL-1249^35^. To differentiate between this M2 binding site as well as the two *docking* poses, potential side chain interactions with cloxyquin and amino acids near the SF (T116, T322, T323), at the M2 (I141, P142, F145, T149) and at the M4 helix (E349, F352, F355, K356, Q359) were subsequently selected for targeted mutational analyses (Fig. 2f, g).

### Mutations around the SF potentiate the agonistic effect

**Fig. 3:**
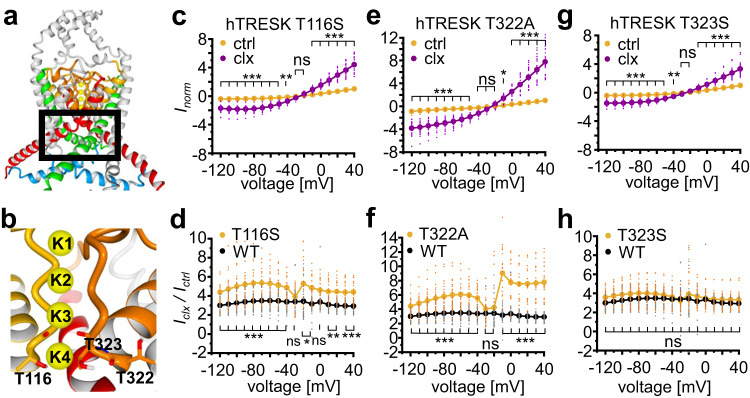
Mutational analysis of SF residues. **a** In silico model of hTRESK. Region analyzed by mutational scan is indicated by black box. **b** hTRESK selectivity filter with amino acids T116, T322 and T323. **c**, **e**, **g** Dot plot and mean ± SEM of *I*_norm_ for mutants hTRESK T116S (c, *n* = 19), T322A (e, *n* = 22) and T323S (g, *n* = 25) in absence (yellow) and presence (purple) of cloxyquin. Currents were normalized for each oocyte to current at +40 mV in absence of cloxyquin. Significance of mean differences was tested by one-way ANOVA and post hoc mean comparison Tukey test and is indicated for each voltage step by ns for *p* > 0.05, * for *p* < 0.05, ** for *p* < 0.01 and *** for *p* < 0.001. **d**, **f**, **h** Dot plot and mean ± SEM of current potentiation *I*_clx_ / *I*_ctrl_ for mutants hTRESK T116S (d, *n* = 19), T322A (f, *n* = 22) and T323S (h, *n* = 25) compared to hTRESK WT (black). Significance of mean differences was tested by one-way ANOVA and post hoc mean comparison Tukey test for each voltage step.

Alanine scanning represents a powerful tool to identify residue side chains that are essential for the TRESK channel activation by cloxyquin. In particular, the exchange of a residue contributing to the cloxyquin-mediated activation to alanine should result in a mutated hTRESK channel with reduced cloxyquin sensitivity. While T116 and T323 directly interact with K^+^ ions in silico, T322 is not part of the inner SF region (Fig. 3a, b). As a compromise of channel function maintenance and mutational analysis, T116 and T323 were replaced by the more homologous amino acid serine instead of alanine. For each mutant the propensity to become activated by cloxyquin was assessed analyzing the normalized currents (*I*_norm_) in absence and presence of cloxyquin. Currents were normalized to the current at +40 mV for each oocyte in absence of cloxyquin (Fig. 3c–h, Supplementary Fig. 3a–c). Next, the effect was quantified by the current potentiation (*I*_clx_ / *I*_ctrl_) and compared to the cloxyquin effect at WT channels.

Here, all three SF mutants showed a current activation (*I*_norm_) in presence of cloxyquin indicating a conserved agonistic effect (Fig. 3c, e, g). Whereas T323S currents were similarly enhanced by cloxyquin as WT currents (Fig. 3h), T116S and T322A surprisingly even exerted significantly enhanced current activation by the compound (Fig. 3d, f, Supplementary Table 3). Noteworthy for T322A, the increased current potentiation is paralleled by strongly reduced currents of the mutant compared to hTRESK WT channels in the absence of cloxyquin (Supplementary Fig. 3b). Therefore, the enhanced cloxyquin effects might be a result from a rescue of the functional loss caused by this mutation. Summarizing the results, direct contribution to the cloxyquin binding site of SF residues T116, T322 and T323 appear less likely since no mutation caused a significant loss of cloxyquin activity. However, the pronounced activation of T116S and T322A could indicate an allosteric contribution. Furthermore, the activity of cloxyquin at T322A is voltage dependent, which is not observed at any other mutant or wildtype channels (Fig. 3f, Supplementary Table 3).

### Amino acids in M2 contribute to the cloxyquin binding site

**Fig. 4:**
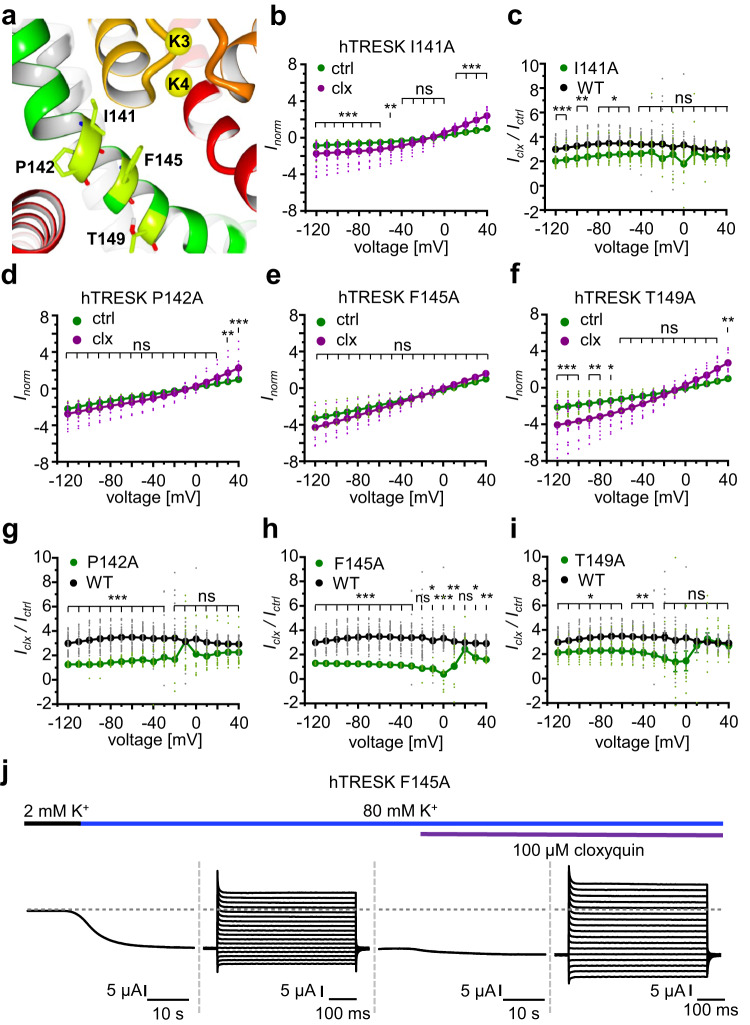
Mutational analysis of M2 residues. **a** hTRESK M2 helix with amino acids I141, P142, F145 and T149. **b**, **d**–**f** Dot plot and mean ± SEM of *I*_norm_ for mutants hTRESK I141A (b, *n* = 28), P142A (d, *n* = 16), F145A (e, *n* = 16) and T149A (f, *n* = 14) in absence (green) and presence (purple) of cloxyquin. Currents were normalized for each oocyte to current at +40 mV in absence of cloxyquin. Significance of mean differences was analyzed by one-way ANOVA and post hoc mean comparison Tukey test and is indicated for each voltage step by ns for *p* > 0.05, * for *p* < 0.05, ** for *p* < 0.01 and *** for *p* < 0.001. **c**, **g**–**i** Dot plot and mean ± SEM of current potentiation *I*_clx_ / *I*_ctrl_ for mutants hTRESK I141A (c, *n* = 28), P142A (g, *n* = 16), F145A (h, *n* = 16) and T149A (i, *n* = 14) compared to hTRESK WT (black). Significance of mean differences was tested by one-way ANOVA and mean comparison Tukey test for each voltage step. **j** Sample trace of hTRESK F145A in absence and presence of 100 µM cloxyquin and 80 mM K^+^.

In silico predictions positioned the M2 amino acids I141, P142 and T149 in spatial proximity to F145 (Fig. 4a)^31^. Thus, all four amino acids were individually mutated to alanine and subsequently analyzed (Fig. 4b–j, Supplementary Table 3, Supplementary Fig. 3d–f). Cloxyquin can activate currents conducted by the M2 mutants I141A and T149A but failed almost completely to activate currents of the P142A mutant (Fig. 4b, d, f, Supplementary Table 3). Only at strongly depolarizing voltages significant current potentiation can be detected for P142A mutant in presence of cloxyquin (Fig. 4d, Supplementary Table 3). However, at these depolarizing voltages, the activity of cloxyquin at P142A mutant does not significantly differ from the cloxyquin activity observed at WT channels (Fig. 4g). In contrast to I141A, P142A and T149A, cloxyquin almost failed to potentiate currents conducted by the F145A mutant (Fig. 4e). Furthermore, cloxyquin activity at F145A mutant significantly differ from activity at WT channels over the full voltage range (Fig. 4h, Supplementary Table 3). Noteworthy, P142A and F145A show strongly altered channel function: While the P142A mutation leads to an almost complete loss of typical hTRESK currents rendering analysis problematic, F145A shows a gain-of-function along with complete loss-of-rectification (Figs. 4d, e, j, Supplementary Fig. 3). In summary, direct interactions can be assumed for F145, which is supported by strong loss of compound activity. For mutants I141A, P142A and T149A, direct interactions seem to be less likely, since current potentiation by cloxyquin and compound effectiveness are not strongly reduced over the full voltage range (Fig. 4).

### Residues of the M4 helix critically contribute to the putative binding site

**Fig. 5:**
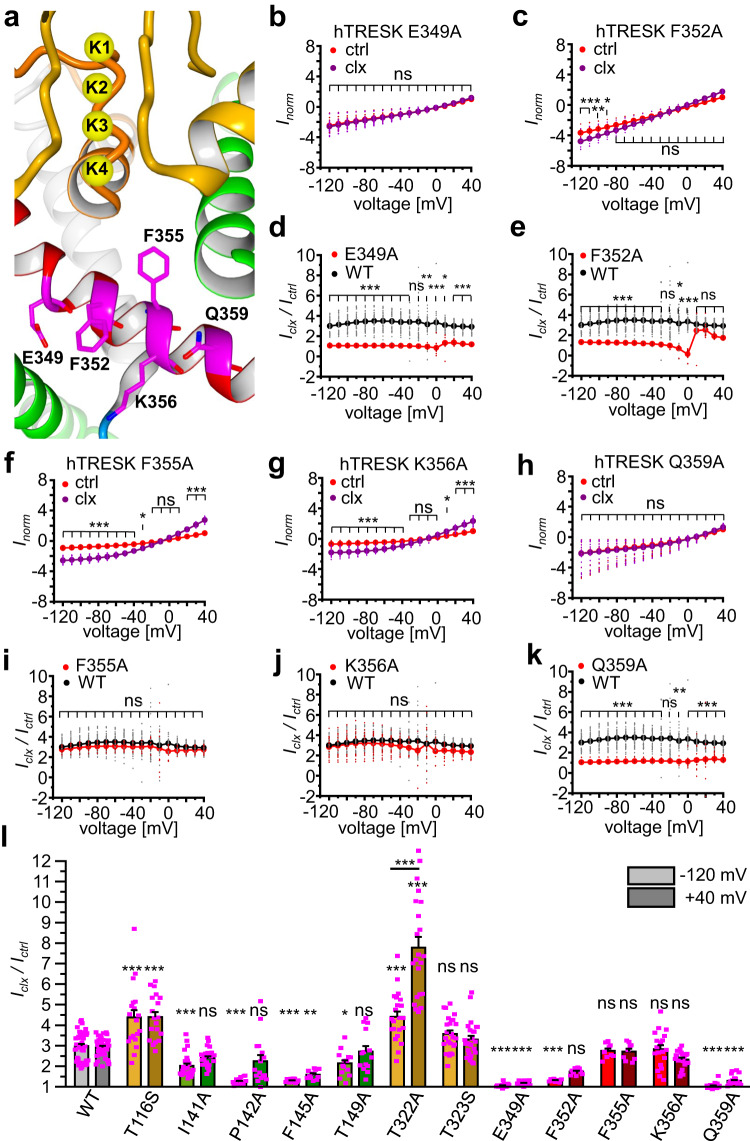
Mutational analyses of M4 residues. **a** hTRESK M4 helix with amino acids E349, F352, F355 K356 and Q359. **b**, **c**, **f**–**h** Dot plot and mean ± SEM of *I*_norm_ for mutants hTRESK E349A (b, *n* = 17), F352A (c, *n* = 9), F355A (f, *n* = 9), K356A (g, *n* = 19) and Q359A (h, *n* = 23) in absence (red) and presence (purple) of cloxyquin. Currents were normalized for each oocyte to current at +40 mV in absence of cloxyquin. Significance of mean differences was analyzed by one-way ANOVA and post hoc mean comparison Tukey test and is indicated for each voltage step by ns for *p* > 0.05, * for *p* < 0.05, ** for *p* < 0.01 and *** for *p* < 0.001. **d**, **e**, **i**–**k** Dot plot and mean ± SEM of current potentiation *I*_clx_ / *I*_ctrl_ for mutants hTRESK E349A (d, *n* = 17), F352A (e, *n* = 9), F355A (i, *n* = 9), K356A (j, *n* = 19) and Q359A (k, *n* = 23) compared to current increment at hTRESK WT (black). Significance of mean differences was tested by one-way ANOVA for each voltage step. **l** Summarized current potentiation *I*_clx_ / *I*_ctrl_ for mutants of SF (yellow), M2 (green) and M4 (red) at −120 mV (lighter color) and +40 mV (darker color) compared to hTRESK WT (gray). Significance of mean differences was analyzed by one-way ANOVA and post hoc mean comparison Tukey test and is indicated as comparison to WT with ns for *p* > 0.05, * for *p* < 0.05, ** for *p* < 0.01 and *** for *p* < 0.001. Numbers of independent oocytes are defined for each mutant and WT in Fig. legends 2–5.

Based on *docking* predictions, M4 residues E349, F352, F355, K356 and Q359 were individually mutated to alanine (Fig. 5a–k). Three of these mutations result in hTRESK channels, which are nearly insensitive to cloxyquin: hTRESK E349A, F352A and Q359A (Fig. 5b–e, h, k, Supplementary Table 3). While normalized currents for F352A were slightly but significantly elevated for more negative voltages, those of E349A and Q359A were almost identical in absence and presence of cloxyquin (Fig. 5b, c, h, Supplementary Fig. 4). Consequently, the current potentiation factors are significantly different for all three mutants compared to WT over a broad voltage range (Fig. 5d, e, k, Supplementary Table 3). On the other hand, mutants F355A and K356A show no significant changes in cloxyquin activity with robust and significant elevated inward and outward currents in presence of cloxyquin (Fig. 5f, g). Furthermore, current potentiation for these mutants is not significantly different to WT channels (Fig. 5i, j, Supplementary Table 3).

In summary, mutational analyses identified several residues, which markedly modulate cloxyquin activity (Fig. 5l). Especially mutations causing pronounced loss of cloxyquin effect indicate a direct interaction between the compound and the amino acid side chain. Most pronounced reductions can be observed for the M2 mutation F145A and the M4 mutations E349A, F352A and Q359A.

### In silico and in vitro analysis identified clox-2 as the binding site

**Fig. 6:**
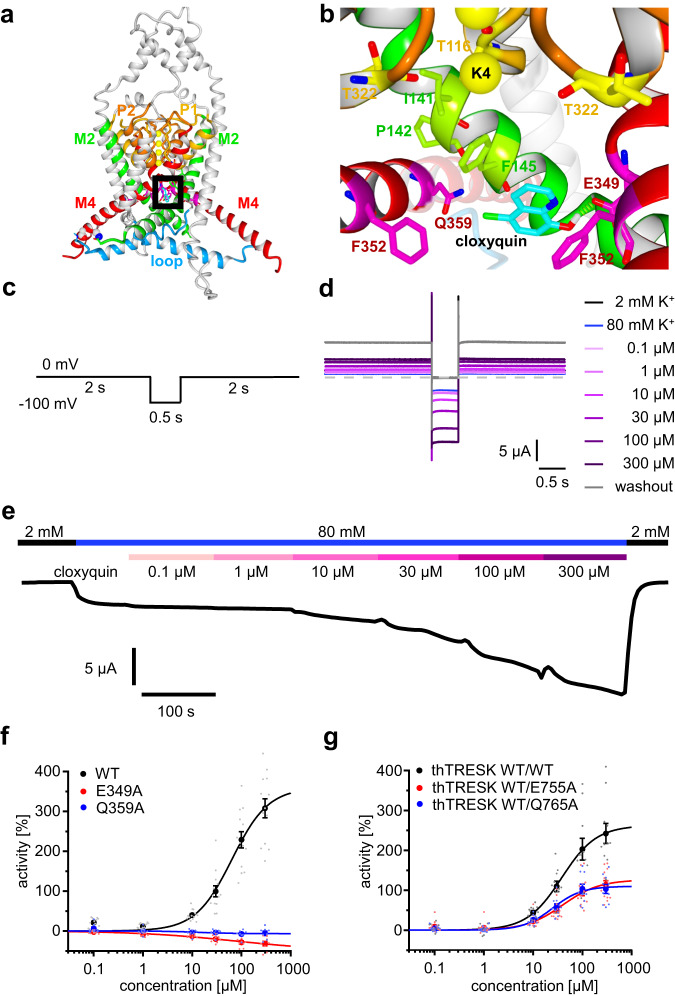
*Docking* pose clox-2 and dose-response relationships of cloxyquin. **a** Binding site localization at the in silico hTRESK model indicated by a black box. **b** *Docking* pose clox-2 was identified as cloxyquin binding site. **c** Pulse protocol for dose response TEVC recordings. The protocol was repeated 195 times with defined application times of different cloxyquin concentrations (see materials and methods). **d** Overlay of final sweeps of all applied solutions containing different concentrations of cloxyquin. **e** Sample line graph generated from repetitive application of pulse protocol from a single oocyte. The line graph was generated from 195 data points and displays the change of current at -100 mV measured every 4.5 s in presence of increasing cloxyquin concentrations. **f** Dot plot, mean ± SEM and dose-response curves for cloxyquin activity evaluated at hTRESK WT (black; *n* = 13), E349A (red; *n* = 9) and Q359A (blue; *n* = 8) expressing oocytes. **g** Dot plot, mean ± SEM and dose-response curves for cloxyquin activity evaluated at thTRESK WT/WT (black; *n* = 11), thTRESK WT/E755A (red; *n* = 13) and thTRESK WT/Q765A (blue; *n* = 10) expressing oocytes.

*Docking* as well as alanine scanning results indicate, that cloxyquin binds to clox-2, since simultaneous interactions between residues F145, E349, F352, Q359 and cloxyquin are not achievable by binding to the M2 or clox-1 binding site (Figs. 2f, 6a, b). To exclude the binding to the other two sites in vitro, we analyzed the activity of cloxyquin in presence of tetrahexylammonium (THA) chloride, which is known for potent TRESK inhibition by binding near the lower SF^15^. *Docking* of THA into the hTRESK / clox-2 complex indicate that the THA binding site overlaps with the clox-1 and M2 but not with the clox-2 binding site (Supplementary Fig. 5a). Previous studies identified a direct competition of BL-1249 with quaternary ammonium ions experimentally by simultaneous application of both compounds causing a right-shift of BL-1249 dose response curve without reduction of maximum agonist efficacy (E_max_)^35^. This reduction of BL-1249 potency (EC_50_) with preserved efficacy (E_max_) was interpreted as a competitive relationship between the two compounds^36^. In case of cloxyquin and THA a different behavior can be detected. Simultaneous cloxyquin application with increasing THA concentrations leads to a prominent reduction of E_max_ while the EC_50_ is unaffected (Supplementary Fig. 5b, Supplementary Table 4). This behavior is known for allosteric relationships between two compounds and therefore excludes the binding of cloxyquin to the clox-1 and the M2 site^36^.

Due to the molecular structure of cloxyquin π/π- or cation/π-interactions with the quinoline system, H-bond interactions with the 8-OH-group as well as halogen-interactions with the 5-Cl-substituent are possible. For the clox-2 binding site *docking* predicts an H-bond between the carboxy moiety of E349 and the 8-OH-group of cloxyquin. Further, an interaction between the 5-Cl-atom and the terminal carbamoyl-group of Q359 can be observed in silico. Similar interactions were previously observed for other arylhalogenes^37^. Since mutations E349A and Q359A lead to a complete loss of agonism, the H-bond as well as the halogen-interaction are identified as most crucial for the agonistic effect. Furthermore, aromatic interactions with amino acids F145 and F352 can be observed, which are also in accordance with results from alanine scan. Interestingly, the predicted binding pose facilitates a coupling of opposing M4 helices by simultaneous interactions with E349 from one and with Q359 from the adjacent M4 helix in silico (Fig. 6b).

### Evaluation of the compound / channel stoichiometry

Since hTRESK is a homodimer, two virtually identical binding sites are present at each channel enabling the possibility of simultaneous binding of two molecules. Consequently, the question is raised if one or two cloxyquin molecules are needed to elicit full stimulatory effect. The maximum efficacy (E_max_) can be used to quantify the ability of cloxyquin to induce a certain response^38^. If occupation of the binding sites is directly proportional to the exerted effect of cloxyquin, E_max_ should be ideally cut in half if only one of the binding sites is functional. On the other hand, if occupation of a single binding site is sufficient to elicit full stimulatory response, E_max_ should be unaffected by a functional knockout of one binding site, which was previously demonstrated for other ion channels with multiple binding sites for the same ligand^39,40^. To address the relevance of compound / channel stoichiometry, dose-response curves were recorded for homodimeric hTRESK WT, E349A and Q359A as well as heterodimeric tandem hTRESK (thTRESK) channels, that allow for a defined channel composition bearing a mutated and a single functional binding site (Fig. 6c–g). At homodimeric WT channels cloxyquin achieves an EC_50_ value of 64 ± 11 µM and a fitted E_max_ of 360 ± 25% (Fig. 6f, Supplementary Table 5). Like previously observed for the mutational analysis with a single concentration, homodimeric E349A and Q359A channels were insensitive to cloxyquin. Therefore, fitting for both mutants result in non-conclusive fitting parameters (Supplementary Table 5). These results also demonstrate that each of the two single mutants can completely disrupt the compound / channel interaction in each binding site. To analyze the effect of only one non-functional binding site we utilized the tandem TRESK channels thTRESK WT/E755A and thTRESK WT/Q765A, that correspond with the mutations E349A and Q359A. The subunits in the tandem constructs are connected by a 22 amino acid linker adapted from previous studies^41^. Since insertion of such a linker can influence channel properties all pharmacological parameters were compared to the cloxyquin activity at the tandem wildtype channel (thTRESK WT/WT). At thTRESK WT/WT cloxyquin achieved an EC_50_ of 38 ± 2 µM and an E_max_ of 262 ± 5% (Fig. 6g, Supplementary Table 5). Compared to these values, the EC_50_ values of cloxyquin at both mutated thTRESK channels are not significantly altered, while the E_max_ values are halved. Interestingly, functional knockout caused by one of the two mutations also abolishes the biphasic current amplification, which was previously observed for hTRESK WT stimulation characterizing the occupation of both binding sites by cloxyquin as a stepwise process (Supplementary Fig. 6). These results lead to the conclusion that the agonistic effect can be partially evoked by occupation of one binding site, while full stimulatory effect is achieved by binding of two cloxyquin molecules.

### hTRESK T322A reduces conductivity in silico by reduction of ion permeation rate

Previous results consistently identified the binding site at the M2/M4 interface according to clox-2 *docking* pose, while binding to clox-1 was excluded due to the non-competitive interaction with THA^35,36^. Thus, hypersensitivity of hTRESK T322A expressing oocytes to cloxyquin might be a result of allosteric contribution of the analyzed residue to the agonistic mechanism. Furthermore, hypersensitivity of T322A expressing oocytes to cloxyquin is accompanied by strongly reduced currents compared to hTRESK WT expressing oocytes in absence of cloxyquin, while application of the compound leads to robust current amplification (Figs. 3f, 5l, Supplementary Fig. 3b). These observations indicate that cloxyquin can compensate the negative effect of T322A on biophysical channel function.

**Fig. 7:**
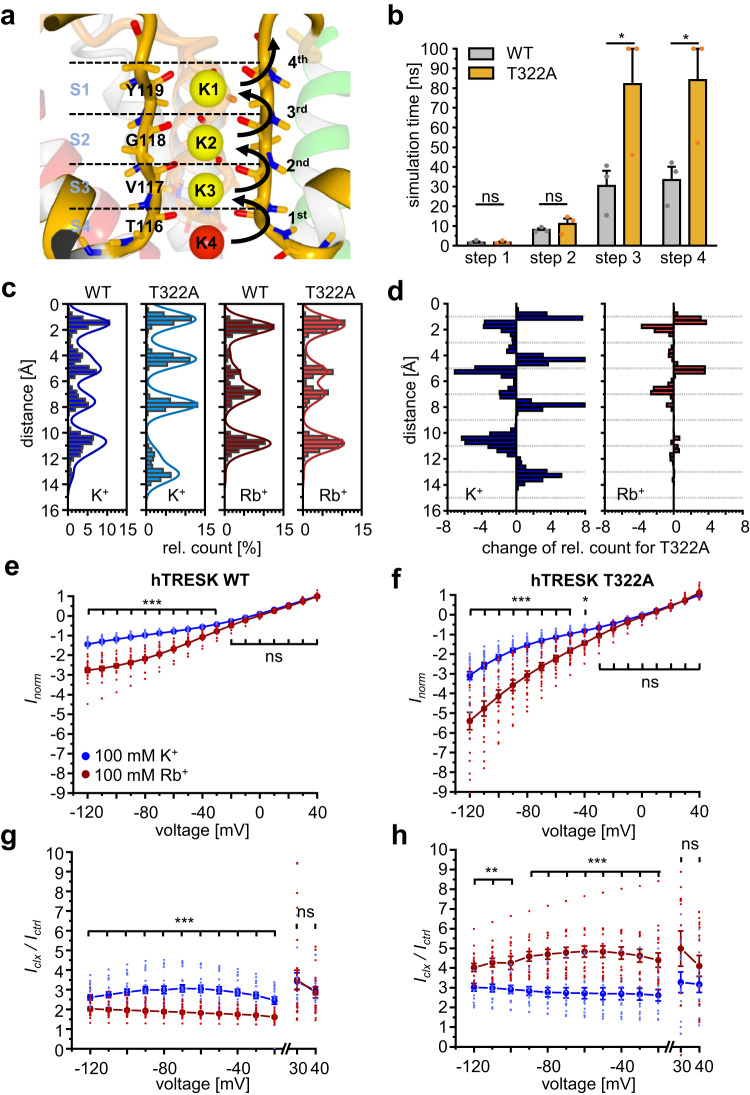
In silico electrophysiology and Rb^+^ measurements. **a** Structure of hTRESK SF1 with backbone of T116 – Y119 forming K^+^ binding sites S1–S4. Binding sites are occupied by K^+^ ions (K1-K3 yellow, K4 red). Permeation time of K4 crossing the SF in four single transitions was evaluated by in silico electrophysiology. **b** Mean occurrence simulation time for 1st, 2nd, 3rd and 4th transition of K4 evaluated in silico. Values are given as mean ± SEM from 3 independent simulations for each variant. Significance of mean differences was analyzed by one-way ANOVA and post hoc mean comparison Tukey test. **c** relative count of ion presence at distinct segments of the SF. Data are generated from accumulated distances for K1/Rb1-K4/Rb4 to Y119 forming the S1 binding site. Distances indicating a leave of SF by K1/Rb1 or K4/Rb4 were excluded from the analysis (Supplementary Table 7). Therefore, histograms are derived from 13992 (WT / K^+^), 17704 (WT / Rb^+^), 13832 (T322A / K^+^) and 13670 (T322A / Rb^+^) single distances. **d** Change of relative count for T322A in comparison to WT. Data were generated from differences of relative count for each of the 50 bins equally distributed from 0 to 16 Å (see materials and methods). Negative values indicate reduced probability of ion presence compared to WT, while positive values indicate higher probability of ion presence for mutant T322A**. e**, **f** Dot plots and mean ± SEM normalized currents (*I*_norm_) for hTRESK WT (e) and T322A (f) expressing oocytes in presence of 100 mM K^+^ (blue) or 100 mM Rb^+^ (red). Currents were normalized for each variant to the mean current generated at +40 mV in presence of 100 mM K^+^. Number of independent oocytes are given in Supplementary Table 8. **g**, **h** Dot plots and mean ± SEM of current potentiation (*I*_clx_ / *I*_ctrl_) at WT and T322A expressing oocytes in presence of 100 µM cloxyquin recorded in 100 mM K^+^ (blue) or 100 mM Rb^+^ (red). Number of independent oocytes are given in Supplementary Table 8. Significance of mean differences for data given in (e–h) was evaluated by two-sided *t* test.

To understand the influence of T322A on channel function in more detail, we applied in silico electrophysiology for WT and T322A channels. The outward conduction process was simulated by membrane-based MD simulations in presence of an electrostatic field (ESF) in three independent simulations for each variant. All simulations were initialized with fully occupied SF carrying four K^+^ ions (K1-K4, Fig. 7a). The transition time needed for the lowest K^+^ ion K4 to cross the SF in four steps was recorded. The simulation time for each replicate was set to 100 ns using the previously equilibrated hTRESK model, which was sufficient in similar studies to display a single ion permeation through the SF^35^. Comparing transition times, K4 transition at hTRESK T322A channels is significantly prolonged (Fig. 7b). In 2 out of 3 T322A simulations, K4 did not achieve the 3rd and 4th transition within 100 ns. On the other hand, simulations of WT hTRESK channel show a mean time of 33.3 ± 6.7 ns to achieve the 4th transition step (Supplementary Table 6).

Under normal conditions, the conduction process through the SF is characterized by smooth energetical differences between the distinct K^+^ binding sites S1–S4 ensuring the mobility of ions in the SF^42,43^. To analyze if the mobility of K^+^ ions is also influenced by T322A without ESF, we performed additional MD simulations (150 ns per replicate) evaluating the mean probability of K^+^ ion presence in distinct segments of the SF (Fig. 7c; see materials and methods). While the overall locations of S1–S4 are not considerably different (peaks in Fig. 7c), the mobility of K^+^ between the individual binding sites is reduced in case of T322A. This can be observed by the reduced presence of K^+^ ions at T322A between the distinct peaks of S1–S4 (Fig. 7c, d).

Although these results might explain strongly reduced currents in TEVC recordings in absence of cloxyquin, they cannot directly explain how the compound is able to compensate the influence of T322A on channel conductivity. Since an allosteric interplay is indicated by the previous results, long-range movement coupling between the binding site located at M4 and the SF1/SF2 might link both channel segments enabling a modulation of distinct filter segments by cloxyquin. Indeed, analyzing the movement correlation by dynamic cross correlation matrices (DCCMs) in the MD simulations revealed strongly coupled movements in silico of M4 and the neighbored SF1/SF2 loops for both channel variants, while the opposing SF1/SF2 loops show no movement coupling with the M4 (Supplementary Fig. 7, see materials and methods). Especially movement of residues near E349 strongly correlate with movements of the neighbored SF1/SF2, rendering a direct impact of the binding site on distinct filter segments plausible.

In summary, T322A alters channel function by K^+^ mobility reduction in silico, which leads to prolonged in silico permeation times of ions through the SF. Further, pronounced M4/SF coupling is indicated by in silico movement correlations of M4 and neighbored SF1/SF2 enabling an allosteric modulation pathway between the binding site and the SF.

### Cloxyquin can compensate T322A effects by allosteric SF modulation

In vitro observed hyperactivation of hTRESK T322A by cloxyquin suggests, that the compound compensates the negative impact of the mutation on channel function. Together with the in silico observed M4/SF coupling this indicates an allosteric modulation of the SF induced by cloxyquin binding. SF modulations by a compound were previously visualized for other K^+^ channels by the usage of different permeating ion species^44^. Some K^+^ channels (KcsA, TWIK-1, K_v_7.1) conduct Rb^+^ ions^12,45,46^. Structural analysis for K^+^ and Rb^+^ at KcsA or mutated K^+^ selective NaK (NaK2K) channels showed clear differences in S1–S4 occupancy, that also alter the transition energies between the ion binding sites shifting the position of highest energetic barriers in an ion dependent manner^47,48^. Consequently, alterations of distinct SF segments by a compound result in different efficiencies depending on the permeating ion. To utilize this approach for hTRESK, similar alterations of S1–S4 occupation and ion permeation for K^+^ / Rb^+^ are required. Therefore, we repeated MD simulations without ESF and exchanged the K^+^ by Rb^+^ (Rb1–Rb4). Interestingly, a quick loss of Rb1 was observed for both channel variants (Supplementary Table 7). After loss of Rb1, Rb2–Rb4 occupy similar positions in the SF previously observed for KcsA (peaks in Fig. 7c)^47^. On the other hand, pronounced differences compared to K^+^ can be observed for both channel variants at the inner filter binding sites S2 / S3 (Fig. 7c, d).

Next, we analyzed currents generated in presence of 100 mM Rb^+^ and 100 mM K^+^ in vitro by TEVC without further stimulation by cloxyquin. At both variants inward currents are significantly elevated for 100 mM Rb^+^, while outward currents are not altered (Fig. 7e, f, Supplementary Table 8).

Since both ion dependent alterations (SF occupation / ion permeation) are observable for both hTRESK variants, we finally analyzed the activity of 100 µM cloxyquin in presence of 100 mM Rb^+^ and 100 mM K^+^. Comparing the ratios of current potentiation (*I*_clx_ / *I*_ctrl_) revealed significant differences in compound activity depending on the permeating ion species (Fig. 7g, h, Supplementary Table 8). Interestingly, the exchange of K^+^ by Rb^+^ affects the activity differently for WT and T322A mutant: While Rb^+^ led to reduction of *I*_clx_ / *I*_ctr*l*_ for the WT, cloxyquin activity at hTRESK T322A is elevated by Rb^+^. Furthermore, alterations of compound efficiency are only significantly altered for inward directed currents supporting that cloxyquin effect is depending on the permeating ion.

### Cloxyquin reduces fraction of inactivating hTRESK channels under hyperpolarization

**Fig. 8:**
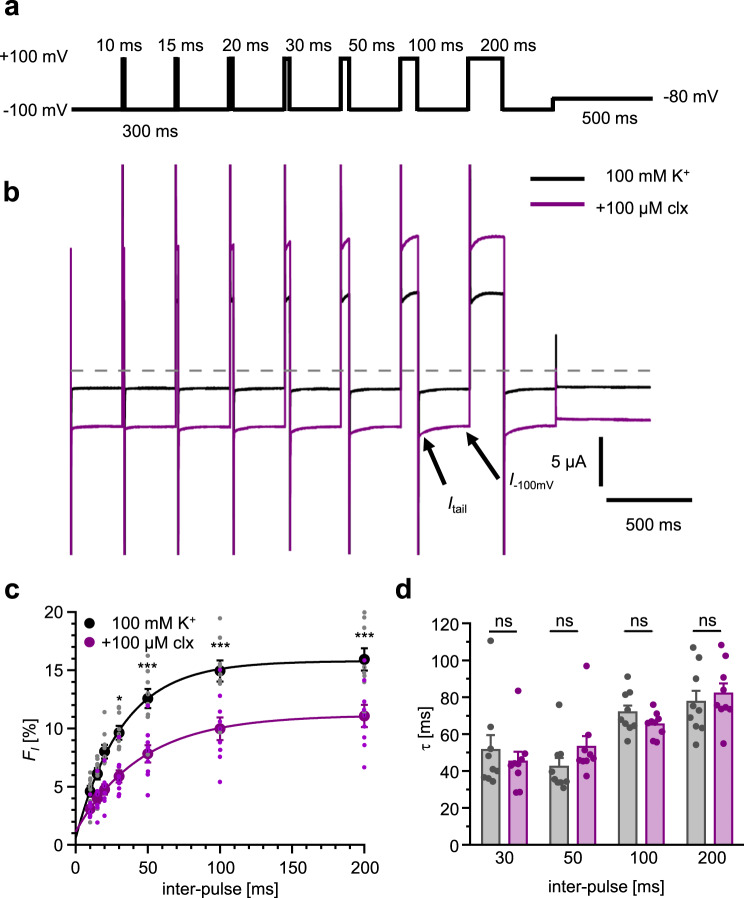
Kinetic analysis of hTRESK WT SF-inactivation. **a** Pulse protocol. The protocol was applied twice per oocyte in absence and presence of 100 µM cloxyquin. Recordings were performed in 100 mM K^+^ solution. **b** Overlay of sample current trace in absent (black) and presence (purple) of 100 µM cloxyquin. Gray line represents 0 µA. **c** Fraction of SF-inactivated channels (*F*_*I*_) ± SEM at −100 mV evaluated for each inter-pulse (10–200 ms) in absence (black) and presence (purple) of 100 µM cloxyquin (clx; *n* = 9). Significance of mean differences was evaluated by one-way ANOVA and post hoc mean comparison Tukey test. **d** Tau values of SF-inactivation ±SEM in absence (black) and presence (purple) of 100 µM cloxyquin (clx) evaluated by one component exponential fit (*n* = 9). Significance of mean differences was evaluated by one-way ANOVA and post hoc mean comparison Tukey test.

Since current potentiation by cloxyquin is depending on the permeating ion, it is plausible, that the molecular mechanism for channel activation is linked to SF modulation. Previous studies suggest that TRESK gating is not controlled by an intracellular gate but exclusively by conformational stability of the SF^19^. Similar observations were also made for most other K_2P_ channels^7^. Consequently, alterations of gating behavior are often linked to conformational alterations in the SF. Recently it was shown that the instability of the SF at K_2P_ channels including TRESK is depending on the ion flux direction creating a partially voltage-dependent SF-inactivation for inward directed currents^32^. This instability can be detected for hTRESK WT by TEVC measurements with fast changes from depolarizing (+100 mV) to hyperpolarizing (−100 mV) voltages in presence of 100 mM K^+^ (Fig. 8a–d)^33^. To quantify the fraction of partial inactivation (*F*_*I*_) the initial tail current (*I*_tail_) was compared to the steady state current (*I*_−100mV_) at the end of the −100 mV pulse (Fig. 8b). In absence of cloxyquin, *F*_*I*_ increases with the duration of the depolarizing inter-pulse in a one exponential fashion to a maximum value of 15.9 ± 2.9% (*n* = 9; SEM) for the 200 ms pulse (Fig. 8c). In presence of cloxyquin *F*_*I*_ is significantly reduced for inter-pulses longer than 30 ms with a maximum of 11.1 ± 2.8% (200 ms; *n* = 9) indicating a reduction of SF-inactivation by around 30%. In contrast to the alteration of *F*_*I*_, cloxyquin does not significantly alter the kinetics of partial inactivation. Independently from the presence of cloxyquin, the SF-inactivation kinetic can be precisely described by a one component exponential fit indicating a single transition between two states. The time constants for this transition in absence and presence of cloxyquin are not significantly different (Fig. 8d). Consequently, cloxyquin can reduce SF-inactivation and therefore stabilizes the conduction process though the SF.

## Discussion

The hTRESK activation by cloxyquin was previously characterized as a direct ion channel stimulation^30,31^. Especially the observation, that other K_2P_ channels are insensitive to cloxyquin exclude secondary stimulation mechanisms by intracellular proteins like protein kinases A or C^31,49–51^. Furthermore, stimulation by cloxyquin was also characterized as independent from the stimulatory Ca^2+^/calcineurin pathway, which is a unique feature of TRESK compared to other K_2P_ channels^31^. Together with our results, these observations render an indirect stimulation by secondary mechanisms as extremely unlikely.

To understand the hTRESK / cloxyquin interaction in more detail, we utilized different techniques including TEVC measurements in *Xenopus laevis* oocytes, which is a well-established, highly robust and versatile method for functional and pharmacological evaluation of membrane proteins^52,53^. Heterologous expression in these oocytes can also provide limited insights into intracellular mechanisms, however, further secondary effects of TRESK channel modulation should be evaluated within more physiological models as previously performed in other studies^27,29,30,54^.

**Fig. 9:**
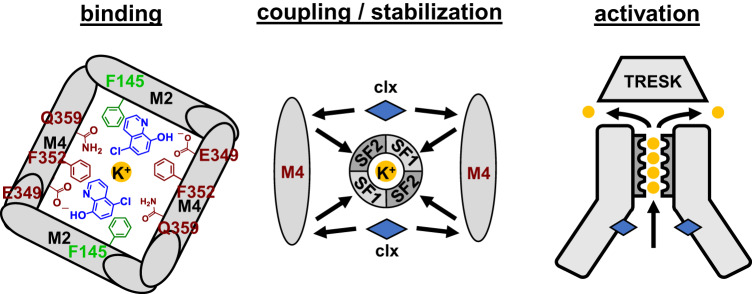
Molecular mechanism of hTRESK channel activation by cloxyquin. Cloxyquin (clx) binds to the M2/M4 helix interface at the cytosolic cavity beneath the selectivity filter. Binding of up to two cloxyquin molecules lead to M4/M4 coupling and subsequent M4/SF coupling. As a consequence of increased coupling, conduction process through the SF is stabilized by prevention of conformational SF collapse.

Our results identified the potential binding site and molecular mechanism for hTRESK activation by cloxyquin (Fig. 9). Structure-activity screening confirmed the importance of the 8-OH- and 5-Cl-substituent for the agonistic effect. Elimination or substitution of both groups result in almost complete loss of activity. Therefore, cloxyquin agonism is exerted synergistically by both substituents as well as the aromatic quinoline scaffold.

In silico binding site prediction at the hTRESK homology model results in projection of two possible binding sites (clox-1, clox-2) as well as the additional binding site near the M2^35^. Mutational analyses as well as competition recordings with THA consistently identified *docking* pose clox-2 as the potential binding site. All interactions predicted by clox-2 are in clear accordance with the structure-activity relationships as well as the influence of the corresponding mutants on cloxyquin activity. Therefore, the molecular mechanism is based on direct binding to the M2/M4 interface through aromatic (F145, F352; quinoline system), H-bond (E349; 8-OH group) and halogen (Q359; 5-Cl-group) interactions (Fig. 9). Interestingly, analyzed residues are identically present in the mTRESK channel supporting the idea of a conserved binding site and agonistic mechanism. Furthermore, hTRESK mutants F145A and F352A show the same gain of function behavior as the mTRESK orthologues F156A and F364A, which hamper the mutational analysis of these residues^31^. Nevertheless, direct interactions between cloxyquin and hTRESK F145 / F352 are in line with the in silico data.

hTRESK E349 and Q359 were identified as the most crucial interaction partners since both alanine mutants repeal the agonism completely. Due to the homodimeric assembly, hTRESK harbors two virtual identical binding sites. Interestingly, E349 and Q359 residues from the same subunit are not part of the same binding site leading to an inter-subunit M4/M4 coupling induced by cloxyquin binding (Fig. 9). This in silico observation is supported by in vitro evaluation of dose-response relationships at homo- and heterodimeric channels. Partial agonistic effects can be elicited by binding of one cloxyquin molecule. However, binding of two molecules is required for full agonistic effect. This is also in line with the biphasic current amplification observed at hTRESK WT and thTRESK WT/WT, which is absent for the mutated tandem channels. Recent studies at different K_2P_ channels showed direct influences of M4 helix movement on the SF altering channel conductance^11,55^. Therefore, reduced efficacy at heterodimers might be mechanistically caused by disturbance of M4/M4 coupling cutting the effect on the SF in half. This hypothesis is supported by the observed movement coupling between M4 and the neighbored but not the opposing SF loops SF1/SF2 modulating only one half of the tetrameric SF. Disturbance of M4/M4 coupling result in reduced M4 modulation and subsequent reduced stabilization of the SF.

Mutational analysis of hTRESK T322 in combination with competition experiments using THA indicate allosteric relationships for cloxyquin and SF residue T322. Using in silico electrophysiology we showed that T322A hampers the ion permeation through the filter by alteration of S1–S4 transition subsequently slowing ion permeation speed through the SF, which is well supported by the low macroscopic currents observed in T322A expressing oocytes in absence of cloxyquin. Since cloxyquin can compensate T322A effects, allosteric impact on the SF by cloxyquin can be assumed, which is in line with previously described M4/SF coupling linking the binding site mechanically to the SF. To verify this hypothesis, we adapted previously described methods for K_v_ channels using different permeating ion species^44^. Since both variants (WT / T322A) can conduct Rb^+^ in vitro and different S1–S4 occupation patterns compared to K^+^ are observed in silico, direct modulation of the SF by the compound can be visualized by ion dependent activity alterations of cloxyquin. Indeed, both variants show significantly altered activities depending on the permeating ion.

Moreover, activities are not only ion but also variant dependent: While usage of Rb^+^ decreased the activity at WT, cloxyquin activity at T322A channels is increased by Rb^+^ suggesting, that cloxyquin exerts its effect by stabilization of the inner filter region around S2 / S3 binding sites. This hypothesis is supported by three different in silico observations: Firstly, Rb^+^ ions show clearly altered ion mobility in the inner filter region (S2 / S3) for each variant, which might explain the different impact on cloxyquin activity in vitro. Secondly, in silico electrophysiology identified the 3rd transition from S2 to S1 as the most crucial transition displayed by strongly elevated transition times for both variants. Consequently, stabilization of S2 could facilitate this transition and lower the energetic barrier. Lastly, pronounced movement correlations for S2 (G118 / G325) and S3 (V117 / I324) with the corresponding M4 helix can be observed facilitating the long-range coupling between the binding site and the inner SF.

Consistent with this, destabilization of the SF is the main reason for hTRESK channel gating and partial SF-inactivation, which can be observed for inward currents at hyperpolarized conditions^32^. Analyses of channel kinetics in absence and presence of cloxyquin show, that cloxyquin can reduce channel inactivation by around 30% shifting the equilibrium to a stable, conductive SF conformation without altered transition kinetics.

Taken together we provide detailed insights into the molecular mechanism of hTRESK channel modulation by cloxyquin. Binding to the M2/M4 interface by direct interaction with F145 (M2) as well as E349, F352 and Q359 (M4) facilitates long range M4/M4 and M4/SF coupling supporting the conductance process and therefore stabilizing a conductive channel state. Identification of the binding site as well as the molecular mechanism of action will be useful for development of specific and highly selective TRESK activators as potential drug candidates for immunomodulation.

## Methods

### Molecular biology, tandem TRESK constructs and oocyte handling

Mutants and cRNAs were generated from hTRESK / pSGEM, which was a gift from Prof. Dr. Erhard Wischmeyer and PD Dr. Frank Döring. Mutagenesis was performed using QuickChange XL II mutagenesis kit (Agilent, Santa Clara, United States). Primers for mutagenesis were purchased from Microsynth AG (Balgach, Switzerland). All mutants were verified by Sanger sequencing. Tandem constructs thTRESK WT/WT / pSGEM, thTRESK WT/E755A / pSGEM and thTRESK WT/Q765A / pSGEM were generated by full gene synthesis (BioCat GmbH, Heidelberg, Germany). The sequence of the tandem constructs is comprised of the hKCNK18 sequence NM_181840.1 and a linker sequence encoding for amino acids AAAGSGGSGGSGGSSGSSGSTG, which is similar to previous utilized linker sequence for other K_2P_ channel tandems^41^. The linker sequence was added in frame to the C-terminus of the DNA sequence encoding for the first hTRESK channel subunit of the tandem followed by another DNA sequence encoding for the second hTRESK channel subunit. To allow for specific mutagenesis, the sequence of the second hTRESK DNA was adapted by silent mutations. cRNAs were generated by in vitro transcription using mMessage mMachine T7 kit and linearized (NheI) cDNA templates. For TEVC measurements, oocytes were purchased from Ecocyte Bioscience (Dortmund, Germany) and injected with 1 ng of wildtype or mutant hTRESK cRNA, except for hTRESK I141A (5 ng), hTRESK P142A (10 ng), hTRESK T322A (5 ng) and the tandem constructs (3 ng). Oocytes were stored at 18 °C for 24–48 h in Bath’s solution containing 88 mM NaCl, 1 mM KCl, 0.4 mM CaCl_2_, 0.33 mM Ca(NO_3_)_2_, 0.6 mM MgSO_4_, 5 mM Tris-HCl, 2.4 mM NaHCO_3_ as well as 80 mg/L theophylline, 63 mg/L penicillin, 40 mg/L streptomycin and 100 mg/L gentamycin.

### Compound and salt solutions for TEVC

With exception of Q09, all quinoline derivatives were commercially purchased from Sigma Aldrich, TCI or Alfa Aesar. Compounds were stored as 100 mM DMSO or 100 mM H_2_O (THA) stocks and freshly diluted from the stock solution for each experiment. For all recordings oocytes were initially superfused with a low concentrated K^+^ solution containing 2 mM KCl, 1.8 mM CaCl_2_, 95.4 mM NaCl and 5 mM HEPES. Depending on the experiment, the concentration of KCl and NaCl was adjusted while CaCl_2_ and HEPES concentrations stayed constant. For compound screening, mutational analysis and dose-response-curves measurement solution contained 80 mM KCl and 17.4 mM NaCl. Analyses of ion permeation were performed with 100 mM KCl or 100 mM RbCl without additional NaCl. For kinetic analyses, measurements were performed in 100 mM K^+^ solution. All final solutions used for TEVC measurements were adjusted to 0.1% or 0.3% (300 µM solutions) DMSO and pH 7.4 using 1 M NaOH. Due to the limits of compound solubility and final DMSO concentration (<0.5%) all measurement solutions were limited to a maximum concentration of 300 µM.

### Two-electrode voltage clamp (TEVC) measurements

Measurements were performed 24–48 h after cRNA injection at room temperature (20 °C) using a Turbo Tec 10CX amplifier (NPI electronic, Tamm, Germany), a NI USB 6221 DA/AD Interface (National Instruments, Austin, USA) and the GePulse Software for data acquisition (Dr. Michael Pusch, Genova, Italy). Recording pipettes were pulled from borosilicate glass and backfilled with 3 M KCl (0.3–1.5 MΩ). Recordings were performed in different solutions (see compound and salt solutions) using two different pulse protocols. For initial compound screening, previous published pulse protocol was adapted and optimized^31^. A repetitive pulse sequence (sweeps) was applied. A single sweep is characterized by a 500 ms pulse to −100 mV every 4 s encompassed by two pulses to 0 mV for 2 s (Fig. 1b). Different test solutions were applied by the following scheme: 2 mM K^+^ solution at sweeps 1–15, 80 mM K^+^ solution without compound at sweeps 16–35, 80 mM K^+^ solution with 100 µM compound at sweeps 36–65, wash out using 80 mM K^+^ solution without compound at sweeps 66–85 and final washout using 2 mM K^+^ solution at sweeps 86–100. Data for dose-response curves were recorded with the same pulse sequence but different application times for the six different compound concentrations: 2 mM K^+^ solution at sweeps 1–15, 80 mM K^+^ solution without compound at sweeps 16–30 followed by 80 mM K^+^ solution with 0.1 µM (sweeps 31–55), 1 µM (sweeps 56–80), 10 µM (sweeps 81–105), 30 µM (sweeps 106–130), 100 µM (sweeps 131–155) and 300 µM cloxyquin (sweeps 156–180). Washout of cloxyquin was performed at sweeps 181–195 with 2 mM K^+^ solution. For mutational analysis as well as Rb^+^ measurements a different sequence of connected pulse protocols was used (Fig. 2a). Initially, a wash in phase changing the perfused solution from 2 mM K^+^ solution to high containing K^+^ (80 mM, 100 mM) or Rb^+^ (100 mM) solution at constant −80 mV was performed for 40 s followed by an IV protocol with 500 ms pulses from −120 to +40 mV in 10 mV increment steps. After the IV protocol phase a second wash-in phase at −80 mV was conducted, where the previously applied solution was exchanged to a solution containing cloxyquin and/or a different permeating ion. After 40 s of wash in the IV protocol phase was repeated. All currents were normalized for each oocyte to the +40 mV pulse in absence of cloxyquin. For kinetic analysis of partial inactivation, the pulse protocol (Fig. 8a) was applied in absence and presence of cloxyquin to the same oocyte. After first application of the pulse protocol in 100 mM K^+^ solution, 100 µM cloxyquin diluted in 100 mM K^+^ solution was washed in at −80 mV for 50 s.

### Synthesis of 5-iodo-quinoline-8-ol (Q09)

Quinolin-8-ol (1.00 g, 6.89 mmol, 1.0 eq), KI (1.22 g, 7.32 mmol, 1.1 eq) and NaOH (285 mg, 7.12 mmol, 1.0 eq) were dissolved in methanol (35 mL). The solution was degassed by bubbling N_2_ through the solution for 30 min at room temperature before cooling to −20 °C. NaOCl (5% in water, 10 mL) was added, leading to a yellow suspension. The mixture was stirred at −30 °C for 30 min. Then, it was equilibrated to pH 7 with 10% HCl. The yellow precipitate was filtered off and recrystallized from methanol and water (80:20). Yellow solid, mp 155 °C), yield 444 mg (24%). C_9_H_6_INO (271.1 g/mol). TLC: Rf = 0.67, strong tailing (cyclohexane/ethyl acetate 67:33). HRMS (APCI): m/z = 271.9575 (calcd. 271.9567 for C_9_H_7_INO + [M + H]^+^). ^1^H NMR (600 MHz, DMSO-D_6_): δ [ppm] = 6.71 (*d*, *J* = 8.3 Hz, 1H, 7-H), 7.56 (*dd*, *J* = 8.5/4.1 Hz, 1H, 3-H), 7.82 (*d*, *J* = 8.3 Hz, 1H, 6-H), 8.16 (*dd*, *J* = 8.5/1.5 Hz, 1H, 4-H), 8.70 (*dd*, *J* = 4.1/1.6 Hz, 1H, 2-H). A signal for the OH proton is not detected in the ^1^H NMR spectrum. ^13^C NMR (151 MHz, DMSO-D_6_): δ [ppm] = 77.3 (1 C, C-5), 114.1 (1 C, C-7), 123.1 (1 C,C-3), 130.4 (1 C, C-4a), 138.5 (1 C, C-6), 139.2 (1 C, C-4), 142.1 (1 C, C-8a), 147.4 (1 C, C-2),159.9 (1 C, C-8). FT-IR: v˜[cm^−1^] = 1558, 1481, 1447 (C=C_arom_), 1362 (O-H bond), 652 (C-I). A signal for the O−H stretching bond is not present in the IR spectrum. Purity (HPLC): 96.8% (t_R_ = 12.62 min). Purity analysis by HPLC was performed as previously described^56,57^.

### In silico generation of hTRESK homology model

The in silico hTRESK model was built using the monomeric *AlphaFold* structure (Uniprot Q7Z418) and the program *YASARA structure* 21 (Supplementary Fig. 2a)^58–60^. For dimerization two monomers were structurally aligned to the crystal structure of structural related TASK-1 channel (PDB ID 6RV3). After alignment, K^+^ ions were added to the four binding sites at the SF and initial clashes of the dimeric hTRESK structure were removed by energy minimization procedure of *YASARA structure* 21 using AMBER15IPQ force field^60,61^. The procedure includes structure cleaning, H-Bond network optimization, generation of surrounding water shell (TIP3P) and pK_a_ prediction (protonation state) of residues at the chosen pH of 7.4^62–64^. To optimize the model and to ensure its stability, the dimer was placed into a membrane surrounded by water molecules and an all-atoms-free MD simulation was performed for protein equilibration over 50 ns using AMBER15IPQ force field, particle-mesh Ewald/Poisson–Boltzmann cutoff 8 Å, periodic simulation cell boundary, long range coulomb forces, 0.9% NaCl, pH 7.4, water density 0.997 (TIP3P water model), 1 atm pressure, a temperature of 298 K and simulation time steps of 2 × 1.25 fs (Supplementary Fig. 2b–g). All parameters were constantly controlled by an NPT ensemble like previously described^65^. After 50 ns an equilibrium of protein movement in the membrane is achieved indicated by a constant RMSD of the whole protein as well as a constant RMSD for the regions of interest (Supplementary Fig. 2c–g). To reduce the influence of the structurally not well characterized intracellular loop as well as the total size of the model, residues between P170 and P254 of the intracellular M2-M3 loop were replaced by variable distance restraints (spring) based on the mean distances between both prolines evaluated at the equilibrium of the initial simulation (Supplementary Fig. 2d, e). Springs were parameterized separately for each monomer. Mean distances for springs were set to *d* = 33 Å (monomer B) and *d* = 40 Å (monomer A) with a flexible border of *d*_delta_ ± 10 Å for both. For all further production MD simulation runs, the model with modified internal loop structure was used.

### *Docking* and Molecular dynamics (MD) simulations

*Docking* and MD simulations were conducted using the optimized hTRESK channel model with flexible distance restraints as a replacement for the intracellular M2-M3 loop (see generation of homology model). *Docking* of cloxyquin into hTRESK channel model was performed using YASARA 21, implemented AutoDock procedure and AMBER15IPQ force field like previously described^56^. Cloxyquin was imported into YASARA using SMILES code followed by structure optimization using semi-empirical quantum mechanics force field (MOPAC). For local *docking*, a simulation box with the size of 18 × 18 × 18 Å was placed around amino acids of the inner cavity. After 100 docking runs, cloxyquin/receptor complexes were sorted by dissociation constants and two separate docking poses (clox-1, clox-2) showing the lowest constant for each conformation were selected. Membrane embedded MD simulation production runs were conducted for 3 × 100 ns (ESF simulations) or 3 × 150 ns (ion occupancy simulations) for each condition with simulation time steps of 2 * 1.25 fs. Simulation parameters are summarized in Supplementary Table 9. To ensure, that the protein and the analyzed properties have equilibrated, we tracked the RMSD for the SF backbone and the SF ions (see Supplementary Figs. 8–11). Since no uncontrolled RMSD drift caused by protein reorientation could be detected, data were analyzed of the complete production run time interval. Data acquisition and analysis were conducted using the AMBER15IPQ force field and implemented YASARA macros md_runmembrane.mcr and md_analyze.mcr. For each production run, the previously equilibrated hTRESK model with flexible restraints was placed individually in a membrane made of phosphatidyl-ethanolamine (PEA) molecules and the complex was initially refined. After initial refinement, water molecules (TIP3P model) were added to the simulation box and a second energy minimization was conducted. With this procedure the membrane and water environment was unique for each production run ensuring slightly altered starting configurations and therefore independency of the analyzed results from the starting configurations. For simulations of the conductance process, an electrostatic field with a constant force of 0.1 Volt/nanometer (0.1 Giganewton / coulomb) directed from intracellular to extracellular space was added to the AMBER15IPQ force field. Temperature and pressure were set to standard parameters of the YASARA macro md_runmembrane.mcr with 298 K and 1 bar. Both parameters were constantly controlled (NPT ensemble) and automatically rescaled like previously described^65^. Simulations were documented by snapshots every 0.1 ns. DCCM were calculated from accumulated data of three independent MD simulations with electrostatic field for each variant using the adapted md_analyze.mcr like previously described^44^. DCCM values indicate the movement correlation of two residues over the complete simulation time and range from -1 (fully anti-correlated) up to +1 (fully correlated). No correlation is indicated by a value of 0. For better visualization, values were transferred to a heat map (See Supplementary Fig. 5).

For analysis of ion mobility (Fig. 7c, d) three independent membrane-based simulations without an electrostatic field were performed for each variant for 150 ns. The distances of all four ions (K1-K4/Rb1-Rb4) to the S1-forming residue Y119 were analyzed every 0.1 ns for the complete simulation time resulting in theoretical number 1500 values per particular ion, 6000 values per simulation and 18,000 values for each condition (variant + K^+^/Rb^+^). Distances of >5 Å between Y119 for K1/Rb1 and T116 for K4/Rb4 indicate a leave SF. Therefore, these values were excluded from the analysis leading to total single distance numbers of 13992 (WT / K^+^), 17704 (WT / Rb^+^), 13832 (T322A / K^+^) and 13670 (T322A / Rb^+^).

### Data analysis

All data were analyzed using OriginPro 2022 (OriginLab, Northampton, USA) and Ana (Dr. Michael Pusch, Genova, Italy). For dose-response curves activity of cloxyquin was calculated and fitted to the Eqs. 1 and 2:

1 $${activity}\,\left[ \% \right]=\left(\frac{{I}_{{clox}}}{{I}_{{ctrl}}}-1\right)* 100$$

2 $${activity}\,\left[ \% \right]=\frac{{E}_{\min }-{E}_{\max }}{1+{\left(\frac{x}{{{EC}}_{50}}\right)}^{h}}+{E}_{\max }$$

E_min_ displays the minimal activity and was fixed to 0 for all fits, while E_max_ displaying the maximum efficiency was derived individually from each data set. The EC_50_ value gives the concentration of half-maximal activation, while the Hill coefficient h gives the slope of the sigmoidal curve. All fit-derived parameters are given as value ± SE in the Supporting Information.

The Fraction of partial inactivation (*F*_*I*_) was calculated using the initial tail current (I_tail_), the steady-state current (I_−100mV_) and the Eq. 3:

3 $${F}_{I}\,[ \% ]=\left(1-\frac{{I}_{-100{mV}}}{{I}_{{tail}}}\right)* 100$$

*F*_*I*_ was separately evaluated for each inter-pulse. The results were fitted to an asymptotic growth function (Eq. 4) in dependence to the duration of the inter-pulse (Fig. 8c):

4 $${F}_{I}=a-b* {c}^{x}$$

a displays the steady-state of *F*_*I*_, b the possible range and c displays the rate of change in dependence of the inter-pulse. Further, the time constant τ of partial inactivation kinetic was determined for each oocyte by fitting the current trace to a first order exponential equation (Eq. 5; Fig. 8d):

5 $$y={a}_{0}+{a}_{1}* {e}^{-\frac{x}{\tau }}$$

a_0_ displays the steady state current, a_1_ the current amplitude and τ the time constant in ms.

### Statistics and reproducibility

All values are given as mean ± SEM. Numbers of independent oocytes, experiments, or simulations are given in the main article, the figure legends or supplementary information with at least three independent replicates. Wherever applicable, data were statistical evaluated by One-way-ANOVA followed by post hoc mean comparison Tukey test or Two-sited *T* test using OriginPro 2022. *P* values are indicated by ns (not significant) for *p* > 0.05, * for *p* < 0.05, ** for *p* < 0.01 and *** for *p* < 0.001.

### Reporting summary

Further information on research design is available in the Nature Portfolio Reporting Summary linked to this article.

## Supplementary information

Supplementary Information

Description of Additional Supplementary Files

Supplementary Data 1

Supplementary Data 2

Reporting Summary

## Data Availability

All relevant data are included in the main article or the Supplementary Information. Source data for the main figures is available as Supplementary Data 1. First and last snapshots of each simulation as .sce file (YASARA scene file; Use YASARA view to open) are available as Supplementary Data 2. Further simulations datasets or any other information can be obtained from the corresponding author upon reasonable request.

## Code availability

All used software are either commercially (YASARA structure 22, OriginPro 2021, Microsoft Excel) or freely (Gepulse, Ana; http://users.ge.ibf.cnr.it/pusch/programs-mik.htm) available. All codes were used as provided by the software. No individual codes were used.
