# Supplementary material for: Cloxyquin activates hTRESK by allosteric modulation of the selectivity filter
Source: Commun Biol. 2023 Jul 18;6:745. doi: 10.1038/s42003-023-05114-4 (PMC10354012; doi:10.1038/s42003-023-05114-4)
Supplement: Supplementary file 1 — Supplementary Information [file 42003_2023_5114_MOESM1_ESM.pdf]

Supplementary Information

Cloxyquin activates hTRESK by allosteric modulation of the selectivity filter

Julian A. Schreiber [1),2),*], Anastasia Derksen[2)], Gunnar Goerges[1)], Sven Schütte[3)], Jasmin Sörgel[2)], Aytug K. Kiper[3)], Nathalie Strutz-Seebohm[1)], Tobias Ruck[4)], Sven G. Meuth[4)], Niels Decher[3)], Guiscard Seebohm[1),5)]

1) Institute for Genetics of Heart Diseases (IfGH), Department of Cardiovascular Medicine, University Hospital Münster, Robert-Koch-Str. 45, Münster, Germany.
2) Westfälische Wilhelms-Universität Münster, Institut für Pharmazeutische und Medizinische Chemie, Corrensstr. 48, Münster, Germany.
3) Institute of Physiology and Pathophysiology, Vegetative Physiology, Philipps-University Marburg, Marburg, Germany.
4) Department of Neurology, Medical Faculty, Heinrich-Heine University, Düsseldorf, Germany.
5) Westfälische Wilhelms-Universität Münster, GRK 2515, Chemical biology of ion channels (Chembion), Münster, Germany.

*Corresponding author:
Dr. Julian Alexander Schreiber
Robert-Koch-Straße 45
D-48149 Münster
Tel. +49-251-835872
j.schreiber@uni-muenster.de

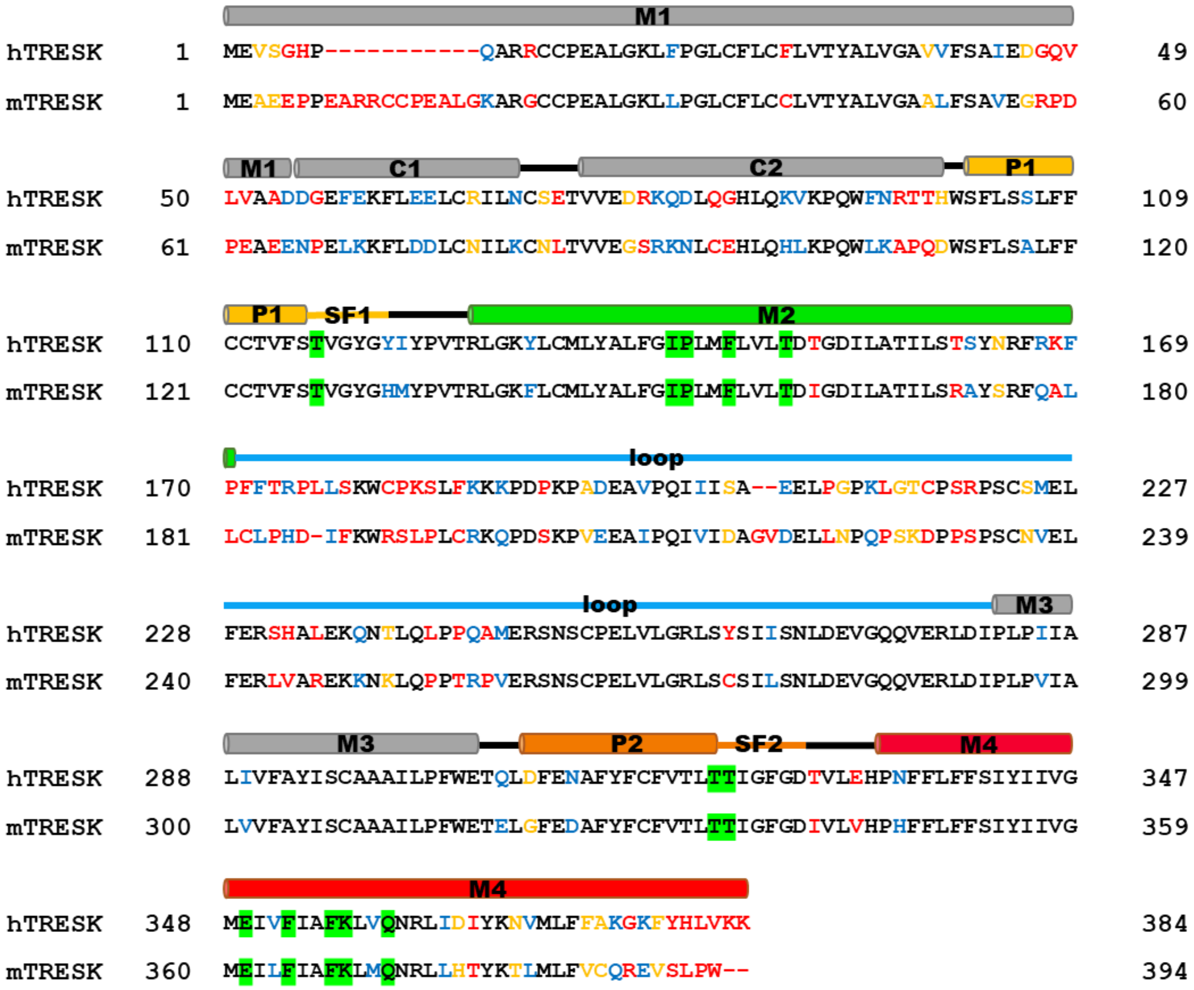

Supplementary Fig. 1 | Protein sequence alignment of hTRESK and mTRESK. Sequences for hTRESK (UniProt Q7Z418) and mTRESK (UniProt Q6VV64) were aligned using Clustal Omega. Sequence identity is 64.5 %. Identical residues are written in black, conserved residues written in blue, semi-conserved written in yellow and non-conserved residues are written in red. Residues, that are used for mutational analyses are marked in green.

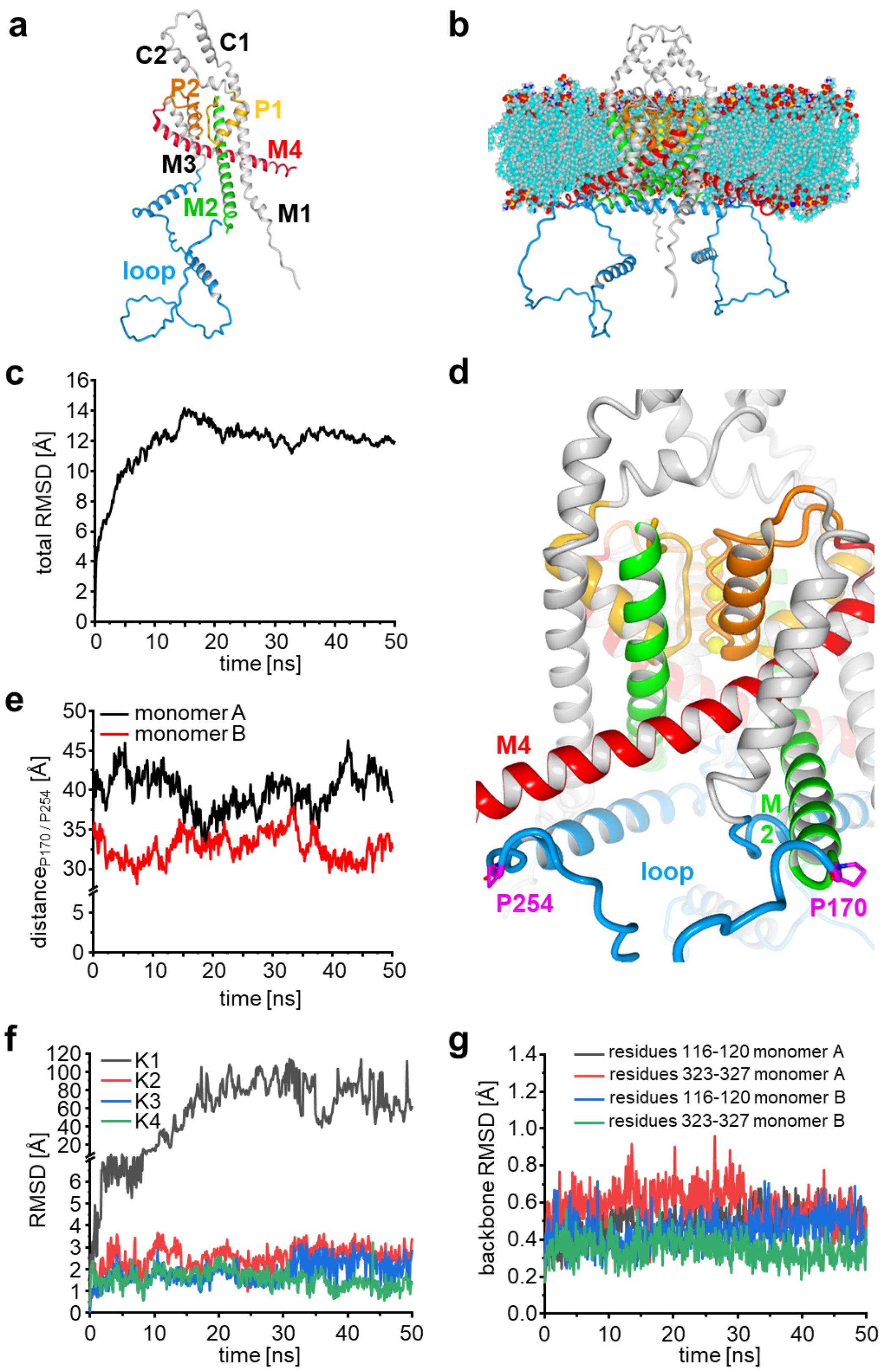

Supplementary Fig. 2 | *In silico* generation of hTRESK model. a monomeric *Alphafold* structure (Uniprot Q7Z418)[1,2]. The monomer can be subdivided into the four transmembrane helices M1-M4, the two extracellular helices C1/C2, the two pore helices P1/P2 and the intracellular loop (blue) between M2 and M3. **b** homodimeric structure of hTRESK model embedded into the membrane. **c** total RMSD

of homodimeric channel derived from backbone movements for a 50 ns MD simulation. **d** close-up depiction of P170 and P254 (magenta). Both residues are located at the end of the structurally not well characterized M2/M3 loop. **e** Distances between P170 and P254 over 50 ns for each monomer. **f** RMSD of K1 – K4 for equilibration simulation of full hTRESK model. High RMSD values of K1 indicate a leaving of the selectivity filter. For production runs, K1 was re-added to the S1 binding site. **g** Backbone RMSD of selectivity filter residues for equilibration MD simulation of full hTRESK model. The constant RMSD values of all four filter segments indicate an equilibration of the protein segments.

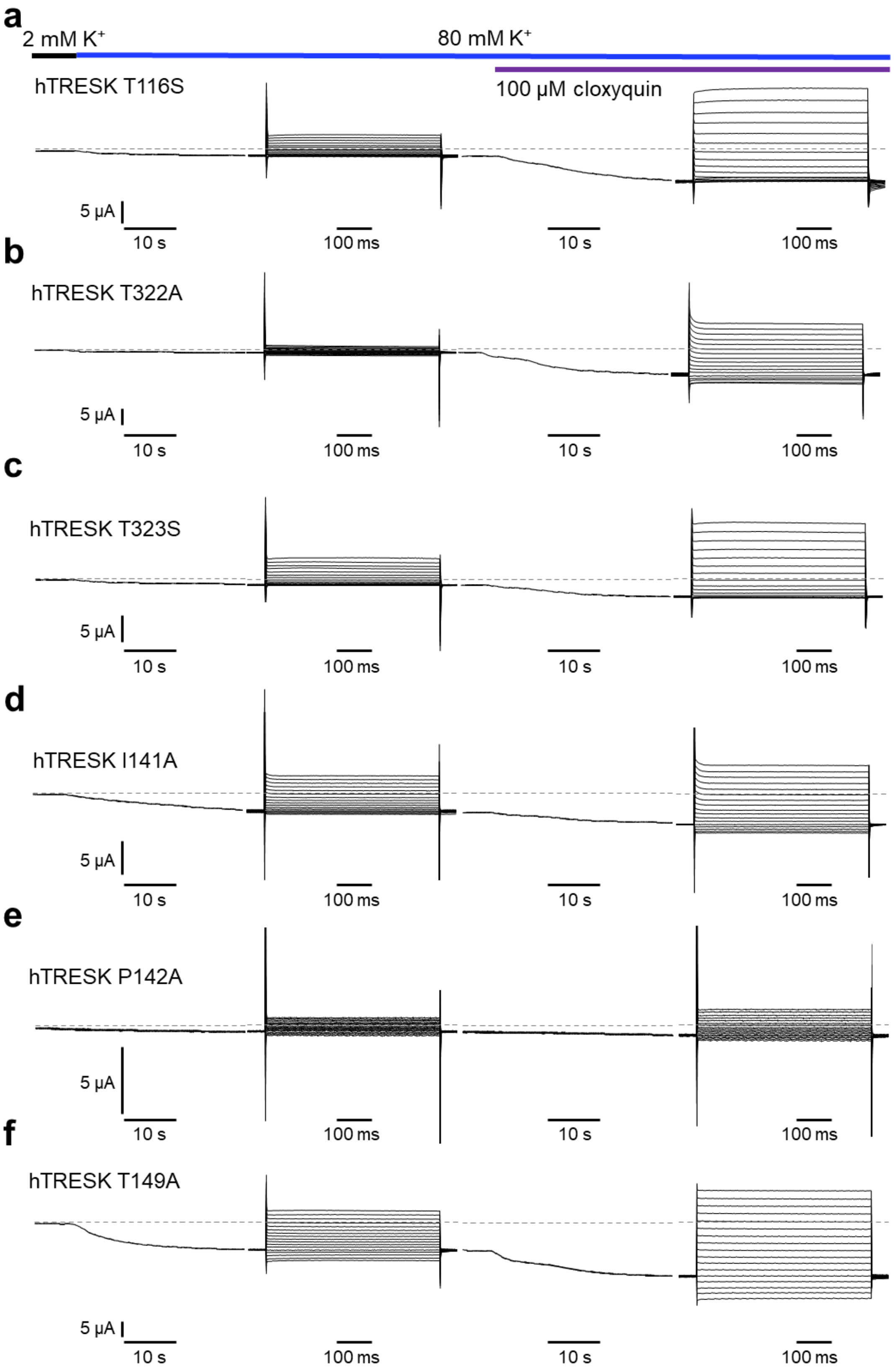

Supplementary Fig. 3 | TEVC sample traces of different SF (a-c) and M2 (d-f) hTRESK mutants.

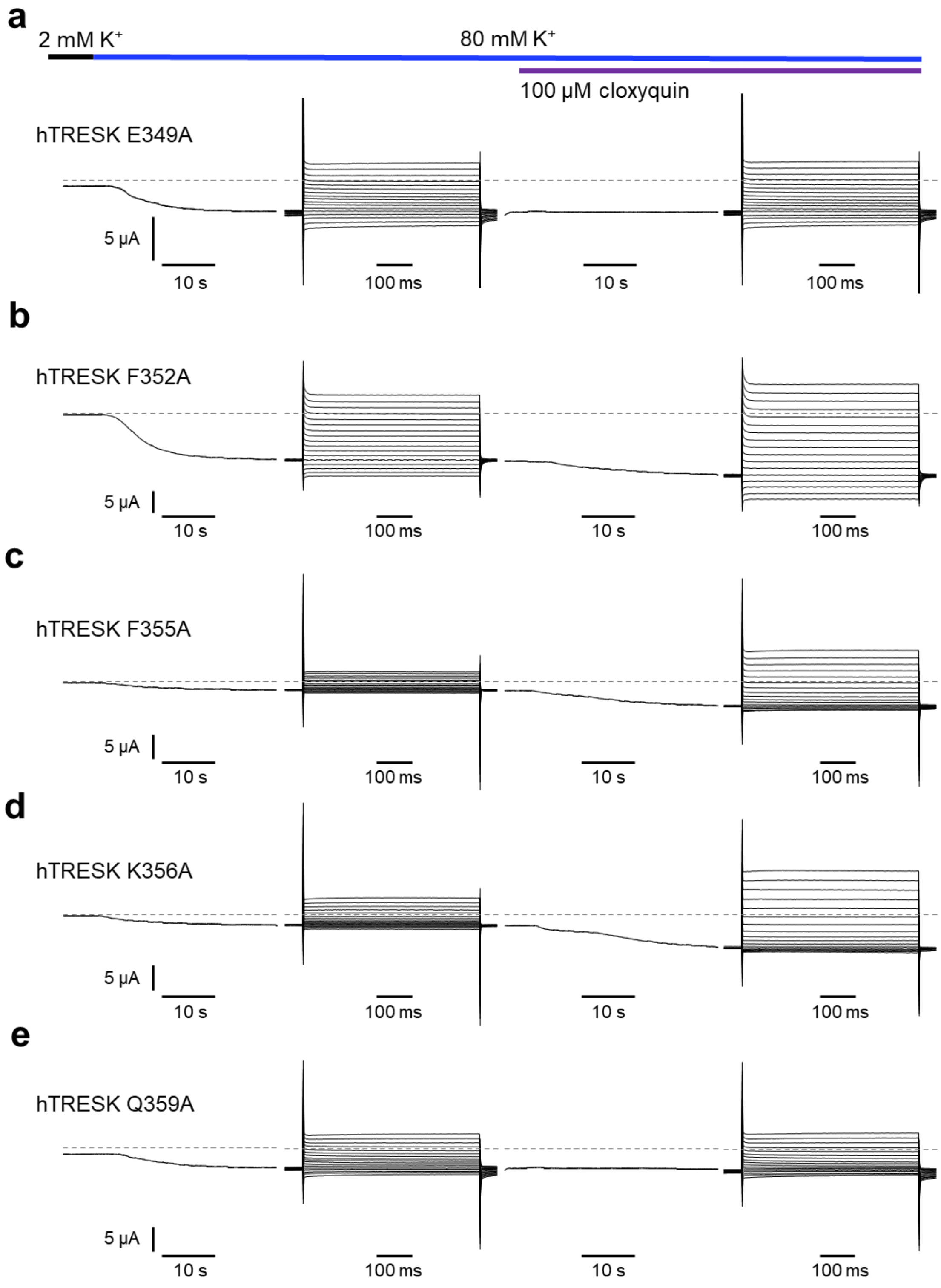

Supplementary Fig. 4 | TEVC sample traces of different M4 hTRESK mutants.

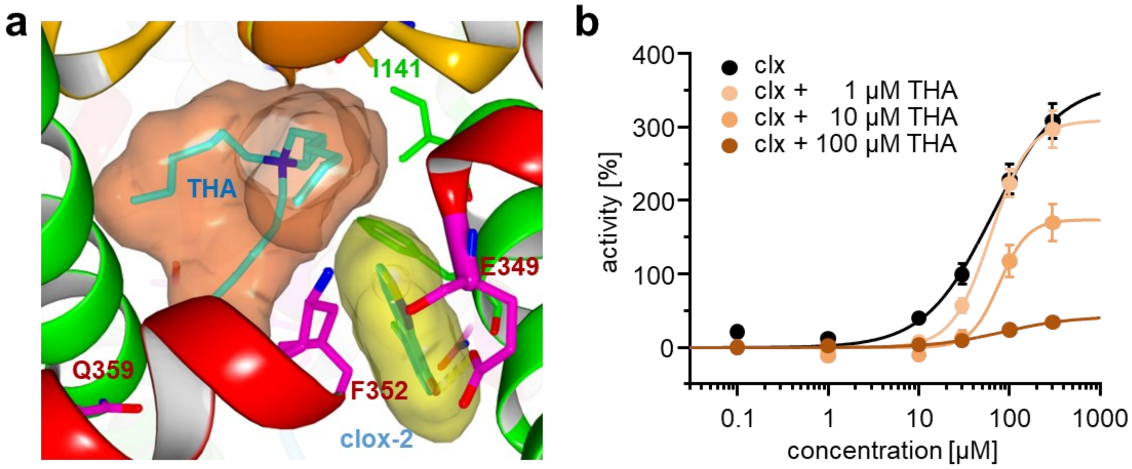

Supplementary Fig. 5 | Non-competitive interaction between tetrahexylammonium (THA) chloride and cloxyquin (clx). a Docking pose of THA at the hTRESK / cloxyquin complex with cloxyquin bound to the clox-2 pose. Molecular surfaces of THA (orange) and cloxyquin (yellow) indicate, that THA binding site does not overlap with clox-2 docking pose. **b** Dose response curves of cloxyquin at hTRESK WT expressing oocytes in presence of different THA concentrations. With increasing concentrations of THA, the maximum efficacy (E_{max}) of cloxyquin is reduced, while potency (EC_{50}) is not significantly altered (Supplementary Table 5) indicating a non-competitive relationship between THA and cloxyquin[3].

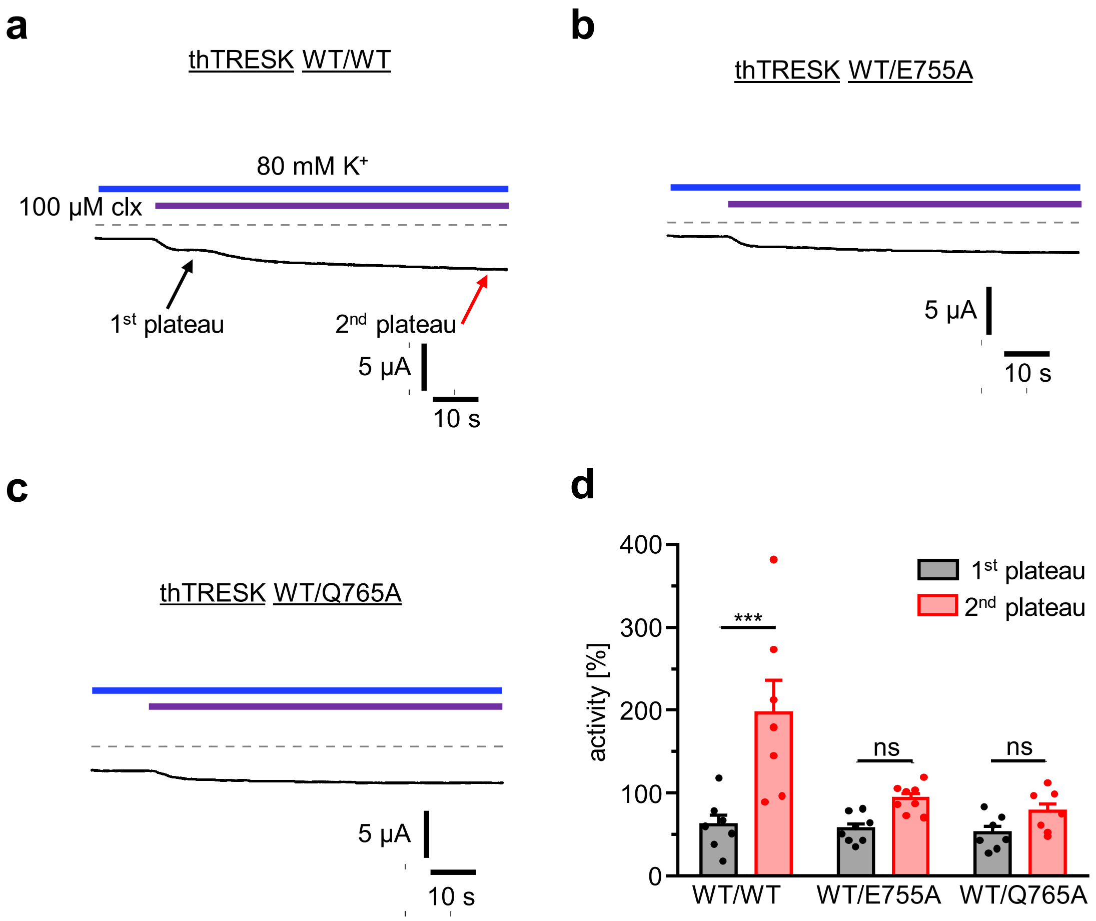

Supplementary Fig. 6 | Biphasic activation of thTRESK by cloxyquin. a-c sample traces of tandem hTRESK expressing oocytes in presence of 80 mM K^+ (blue) and 100 µM cloxyquin (purple). **d** Channel activation caused by 100 µM cloxyquin evaluated at the end of the first (black; ≈10-20 s) and second plateau (red). Significance of mean differences was evaluated by One-way ANOVA followed by post hoc mean comparison Tukey Test and is indicated by *** for $p < 0.001$ and ns for $p > 0.05$.

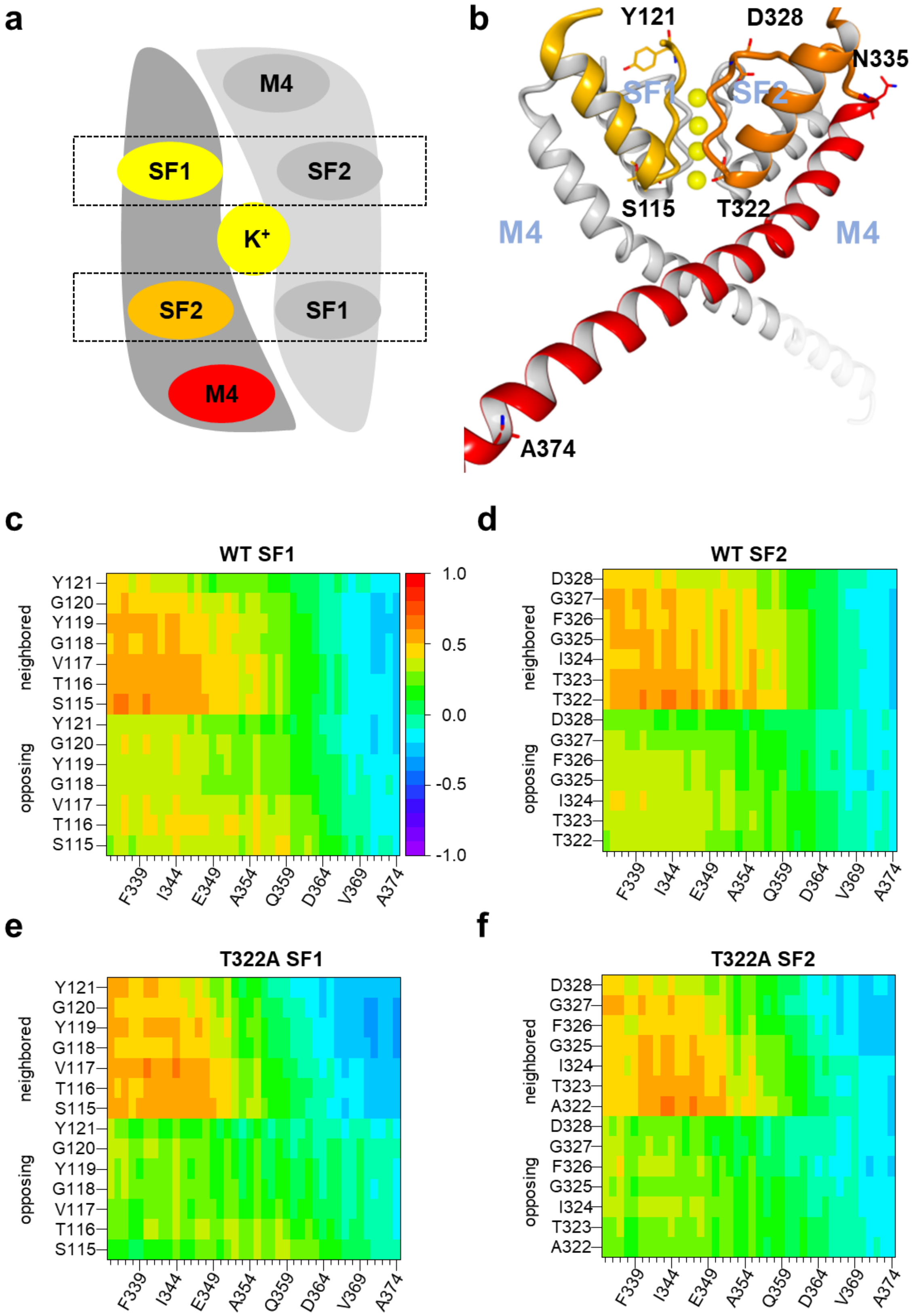

Supplementary Fig. 7 | Dynamic cross correlation matrices (DCCM) for SF1/SF2 coupling with M4. a, b schematic depiction of homodimeric assembly to form the hTRESK channel. Each monomer possesses one M4 helix, one SF1 and one SF2 loop. Each M4 helix of one subunit is neighbored to the

SF2 loop of the same and to the SF1 loop of the second monomer. Consequently, SF1 loop of the same and SF2 loop of the second monomer are opposed to the M4 helix. **d-f** Dynamic cross correlation matrices (DCCM) for M4/SF1 and M4/SF2 coupling of WT (c, d) and T322A channels. Correlations are derived from 3 independent *in silico* electrophysiological simulations (100 ns) for each variant. Heat maps are generated from mean correlation values from these three independent simulations per variant as well as a mean of correlations for both M4 helices present in each simulation. Colors of heat map reflect the degree of correlation from fully correlating (+1.0, red) to non-correlating (0; green) or to fully anti-correlating (-1.0; blue).

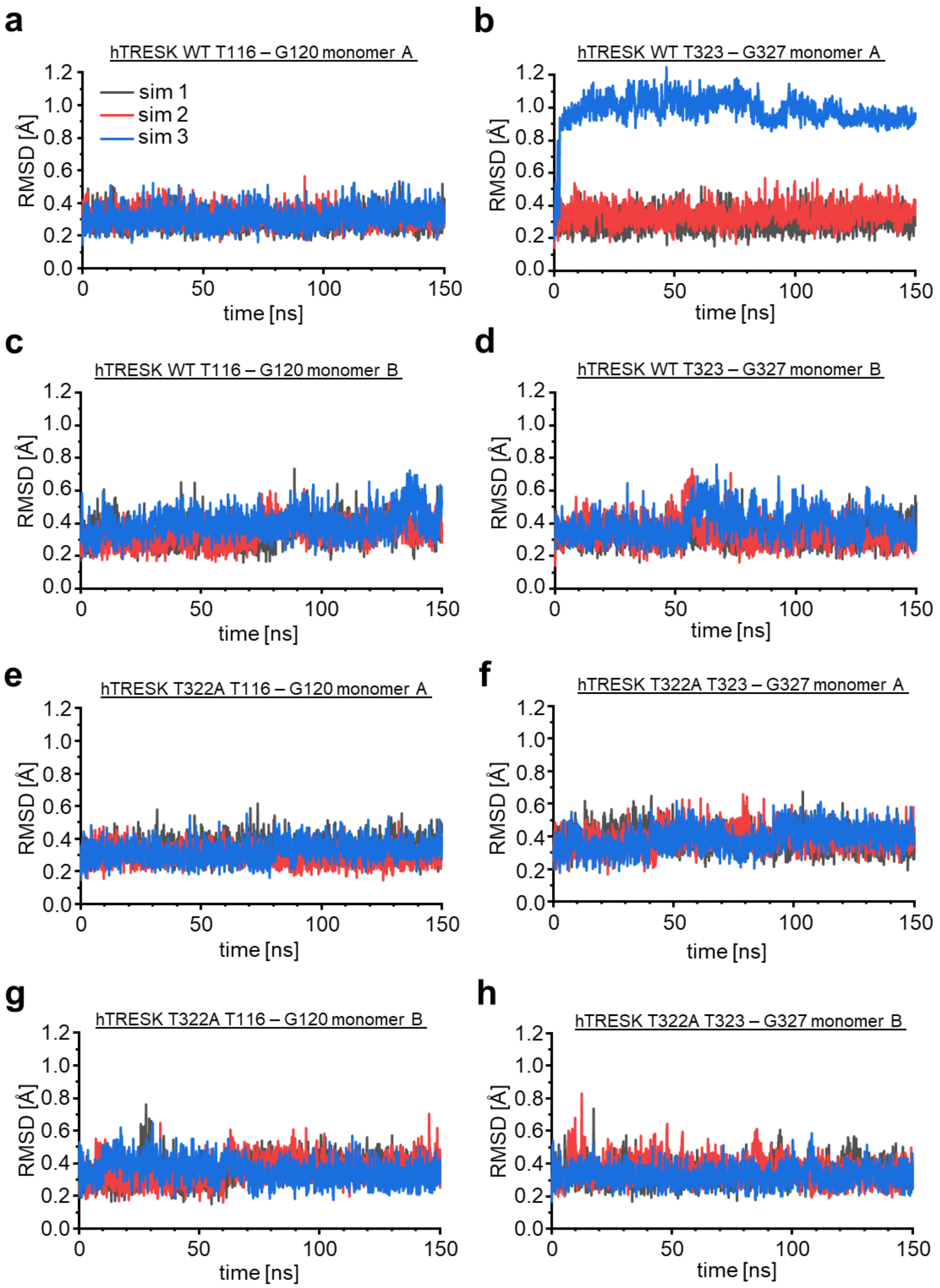

Supplementary Fig. 8 | Selectivity filter RMSD for MD simulations with K^+. a-h Backbone RMSD of selectivity filter residues T116-G120 (SF1) and T323-G327 (SF2) of channel monomers A and B for production runs of MD simulations without electrostatic field to evaluate the mean K^+ ion positions within the selectivity filter. RMSD data are given for simulation 1 (black), 2 (red) and 3 (blue) of each condition. All RMSD values indicate, that MD simulations reached a steady state.

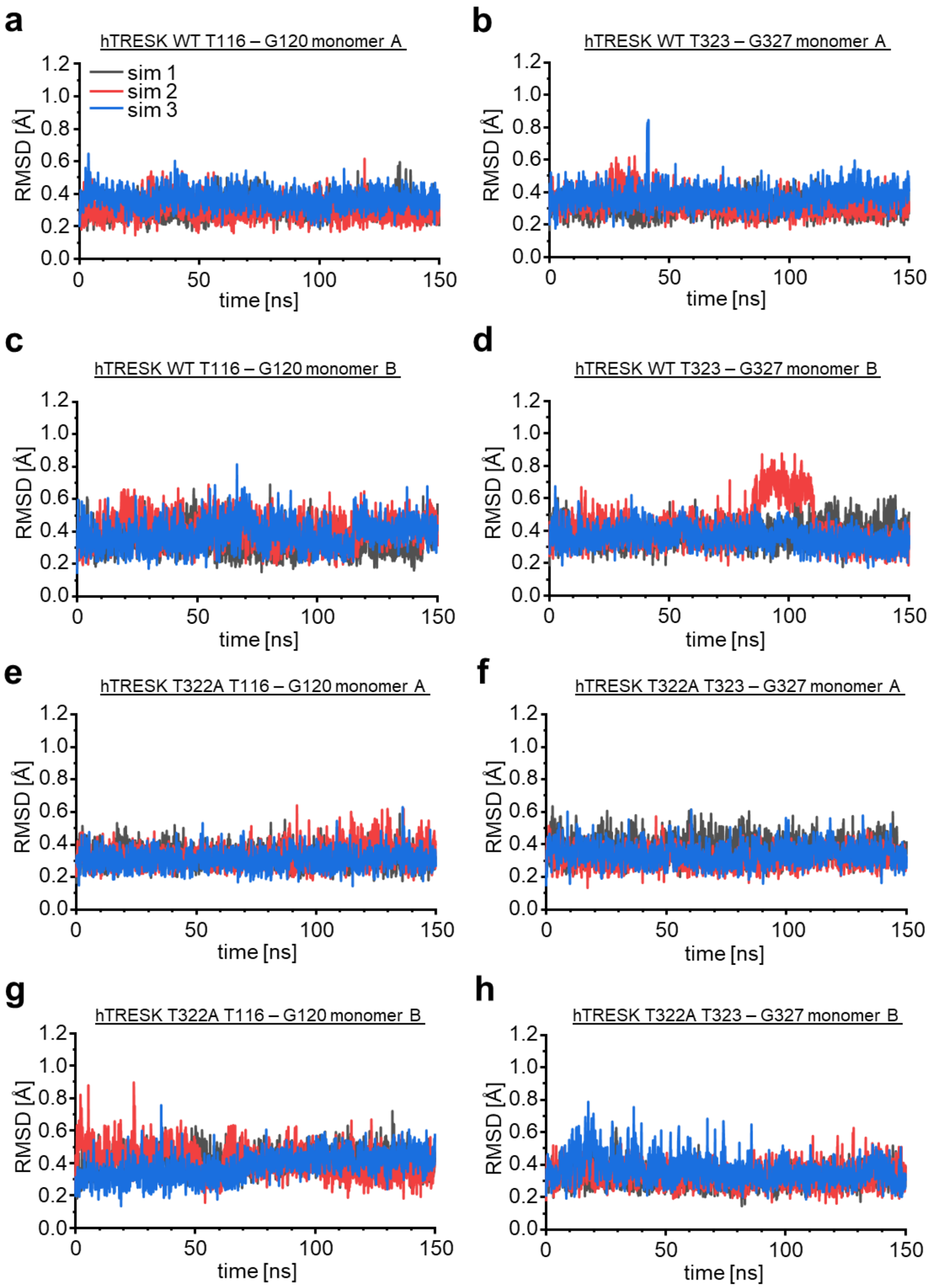

Supplementary Fig. 9 | Selectivity filter RMSD for MD simulations with Rb^+. a-h Backbone RMSD of selectivity filter residues T116-G120 (SF1) and T323-G327 (SF2) of channel monomers A and B for production runs of MD simulations without electrostatic field to evaluate the mean Rb^+ ion positions within the selectivity filter. RMSD data are given for simulation 1 (black), 2 (red) and 3 (blue) of each condition. All RMSD values indicate, that MD simulations reached a steady state.

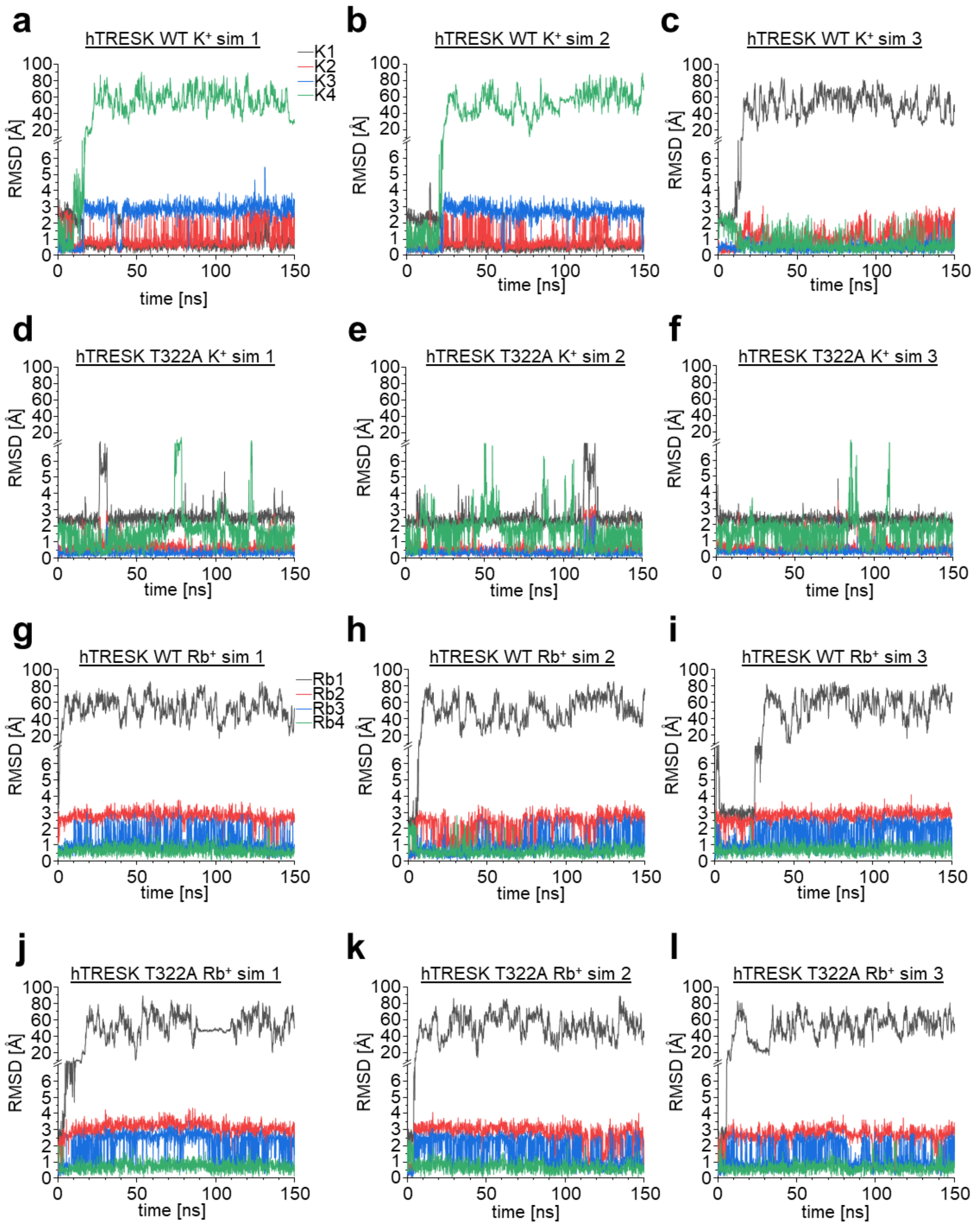

Supplementary Fig. 10 | RMSD of ions K1-K4 / Rb1-Rb4. a-l RMSD values of selectivity filter ions K1/Rb1 (black), K2/Rb2 (red), K3/Rb3 (blue) and K4/Rb4 (green) for each condition and production run of MD simulations for evaluation of the mean ion occupation in the selectivity filter.

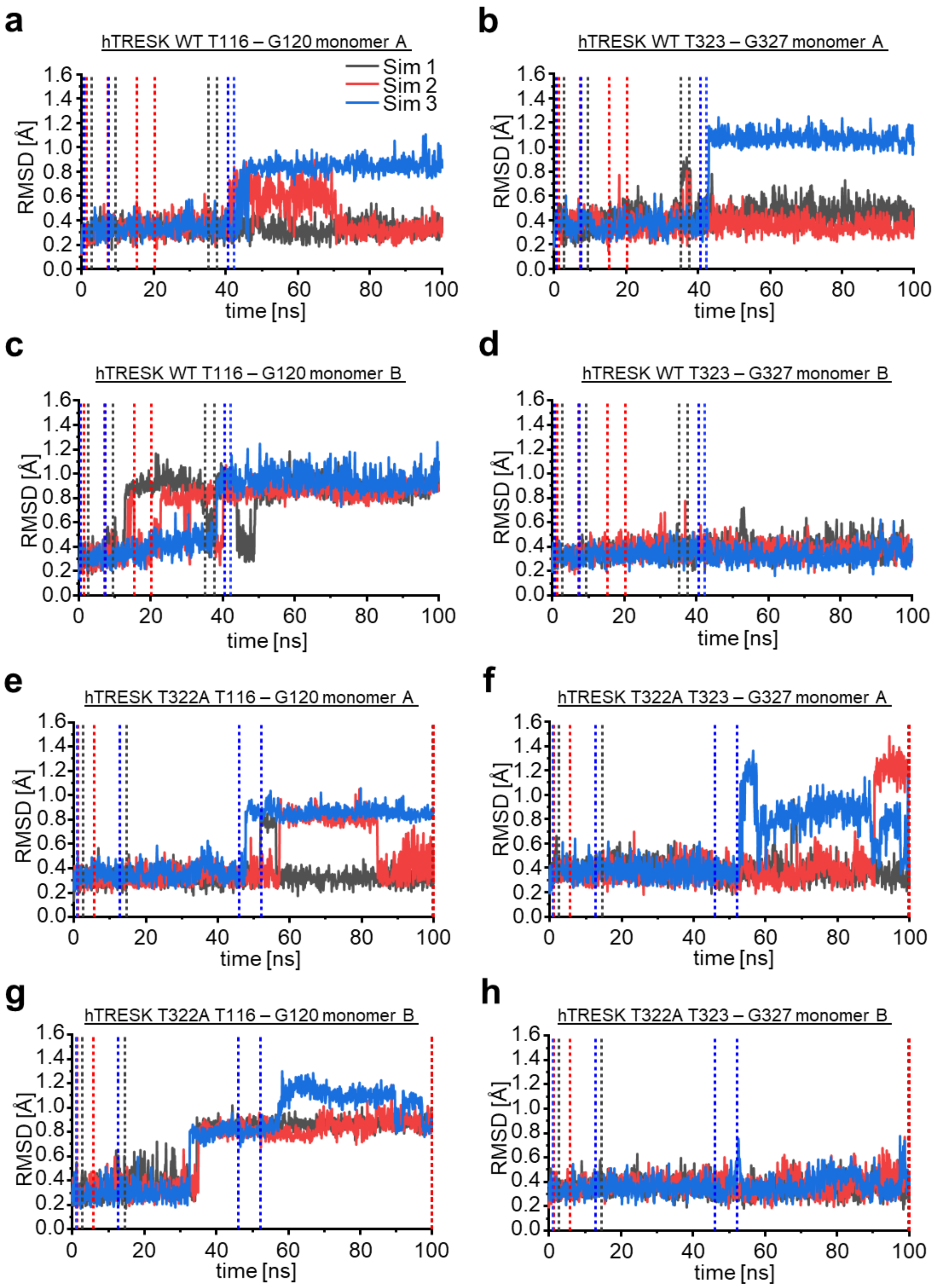

Supplementary Fig. 11 | Selectivity filter RMSD for MD simulations under influence of the electrostatic field (ESF). a-h RMSD values of selectivity filter residues T116-G120 and T323-G327 of monomers A and B for each condition and replicate (simulation 1 black, simulation 2 red, simulation 3 blue). ESF-induced selectivity filter leave of ions are indicated for each simulation by dotted lines of the same color.

Supplementary Table 1 | Mean current potentiation (I_{clx} / I_{ctrl}) determined by 100 µM screening at hTRESK WT expressing oocytes. Values derived from n independent oocytes are given for each compound as mean ± SEM.

compound	I_{clx} / I_{ctrl}	n
cloxyquin	3.75 ± 0.21	5
nitroxoline	1.15 ± 0.01	5
Q01	1.28 ± 0.05	5
Q02	1.68 ± 0.04	5
Q03	1.10 ± 0.02	5
Q04	4.25 ± 0.23	5
Q05	1.32 ± 0.05	5
Q06	1.16 ± 0.04	5
Q07	1.34 ± 0.02	5
Q08	1.11 ± 0.03	5
Q09	2.45 ± 0.26	5
Q10	1.75 ± 0.07	5
Q11	1.50 ± 0.06	5
Q12	1.22 ± 0.01	5
Q13	2.40 ± 0.13	5
Q14	1.61 ± 0.05	5
Q15	1.12 ± 0.03	5

Supplementary Table 2 | Significance of mean differences for 100 µM compound screening. Significance of mean differences for Q01-Q15, nitroxoline (ntx) and cloxyquin (clx) are indicated by ns for p values > 0.05, * for p < 0.05, ** for p < 0.01 and *** for p < 0.001.

	ntx	Q01	Q02	Q03	Q04	Q05	Q06	Q07	Q08	Q09	Q10	Q11	Q12	Q13	Q14	Q15	clx
**ntx**	-	ns	ns	ns	***	ns	ns	ns	ns	***	*	ns	ns	***	ns	ns	***
**Q01**	ns	-	ns	ns	***	ns	ns	ns	ns	***	ns	ns	ns	***	ns	ns	***
**Q02**	ns	ns	-	*	***	ns	ns	ns	*	***	ns	ns	ns	**	ns	*	***
**Q03**	ns	ns	*	-	***	ns	ns	ns	ns	***	**	ns	ns	***	ns	ns	***
**Q04**	***	***	***	***	-	***	***	***	***	***	***	***	***	***	***	***	ns
**Q05**	ns	ns	ns	ns	***	-	ns	ns	ns	***	ns	ns	ns	***	ns	ns	***
**Q06**	ns	ns	ns	ns	***	ns	-	ns	ns	***	*	ns	ns	***	ns	ns	***
**Q07**	ns	ns	ns	ns	***	ns	ns	-	ns	***	ns	ns	ns	***	ns	ns	***
**Q08**	ns	ns	*	ns	***	ns	ns	ns	-	***	**	ns	ns	***	ns	ns	***
**Q09**	***	***	***	***	***	***	***	***	***	-	**	***	***	ns	***	***	***
**Q10**	*	ns	ns	**	***	ns	*	ns	**	**	-	ns	ns	**	ns	*	***
**Q11**	ns	ns	ns	ns	***	ns	ns	ns	ns	***	ns	-	ns	***	ns	ns	***
**Q12**	ns	ns	ns	ns	***	ns	ns	ns	ns	***	ns	ns	-	***	ns	ns	***
**Q13**	***	***	**	***	***	***	***	***	***	ns	**	***	***	-	***	***	***
**Q14**	ns	ns	ns	ns	***	ns	ns	ns	ns	***	ns	ns	ns	***	-	ns	***
**Q15**	ns	ns	*	ns	***	ns	ns	ns	ns	***	*	ns	ns	***	ns	-	***
**clx**	***	***	***	***	ns	***	***	***	***	***	***	***	***	***	***	***	-

Supplementary Table 3 | Mean currents and current potentiation elicit at WT / mutant hTRESK expressing oocytes. All values are given as mean ± SEM. Currents in absence of cloxyquin (clx) at -120 mV and +40 mV are given in µA, while current potentiation (I_{clx} / I_{ctrl}) values have no dimension. For each hTRESK variant number of independent oocytes (n) is noticed.

**hTRESK variant**	**current in absence of clox [µA]**		**current potentiation I_{clx} / I_{ctrl}**		**n**
	**-120 mV**	**+40 mV**	**-120 mV**	**+40 mV**	
**WT**	-2.57 ± 0.43	3.98 ± 0.22	2.98 ± 0.11	2.92 ± 0.07	39
**T116S**	-1.28 ± 0.13	3.08 ± 0.21	4.37 ± 0.36	4.39 ± 0.25	19
**I141A**	-1.92 ± 0.23	2.32 ± 0.20	2.03 ± 0.10	2.40 ± 0.08	28
**P142A**	-1.13 ± 0.09	0.53 ± 0.04	1.25 ± 0.04	2.25 ± 0.29	16
**F145A**	-18.71 ± 1.88	6.49 ± 1.12	1.29 ± 0.03	1.59 ± 0.07	11
**T149A**	-8.90 ± 2.14	4.14 ± 0.76	2.14 ± 0.16	2.72 ± 0.27	14
**T322A**	-0.95 ± 0.11	1.04 ± 0.10	4.41 ± 0.25	7.78 ± 0.52	22
**T323S**	-1.50 ± 0.14	3.49 ± 0.24	3.58 ± 0.16	3.31 ± 0.18	25
**E349A**	-4.23 ± 0.42	1.78 ± 0.18	1.05 ± 0.02	1.17 ± 0.03	17
**F352A**	-18.74 ± 1.71	5.30 ± 0.76	1.31 ± 0.02	1.74 ± 0.05	9
**F355A**	-2.35 ± 0.30	2.42 ± 0.24	2.75 ± 0.12	2.72 ± 0.14	9
**K356A**	-2.51 ± 0.20	3.95 ± 0.42	2.83 ± 0.20	2.32 ± 0.10	19
**Q359A**	-3.49 ± 0.31	2.33 ± 0.28	1.04 ± 0.03	1.28 ± 0.05	23

Supplementary Table 4 | Fitting parameters of dose-response curves for cloxyquin in presence of different tetrahexylammonium chloride (THA) concentrations. Fitting parameters were derived from logistic fits (see materials and methods) of recorded dose-response relationships for cloxyquin at hTRESK WT expressing oocytes. Number (n) of independent oocytes is given for each channel variant. Values for concentration of half maximal activation (EC_{50}), maximum activation (E_{max}) and Hill-coefficient are given as mean ± SE derived from free logistic fitting.

**THA concentration**	**EC_{50} [µM]**	**E_{max} [%]**	**Hill**	**n**
**-**	64.2 ± 10.9	359.6 ± 25.3	1.17 ± 0.14	13
**1 µM THA**	62.5 ± 2.5	309.3 ± 6.5	2.03 ± 0.11	11
**10 µM THA**	76.4 ± 10.5	173.9 ± 14.2	2.75 ± 0.92	10
**100 µM THA**	85.5 ± 36.4	43.2 ± 7.9	1.13 ± 0.28	6

Supplementary Table 5 | Fitting parameters of dose-response curves at different homo- / heterodimeric hTRESK channels. Fitting parameters were derived from logistic fits (see materials and methods) of recorded dose-response relationships at homodimeric human (hTRESK) or heterodimeric tandem human channels (thTRESK). Number (n) of independent oocytes is given for each channel variant**.** Values for concentration of half maximal activation (EC_{50}), maximum activation (E_{max}) and Hill-coefficient are given as mean ± SE derived from free logistic fitting.

**Channel variant**	**EC_{50} [µM]**	**E_{max} [%]**	**Hill**	**n**
**hTRESK WT**	64.2 ± 10.9	359.6 ± 25.3	1.17 ± 0.14	13
**hTRESK E349A**	83.4 ± 214.3	-50.9 ± 26.3	0.43 ± 0.17	9
**hTRESK Q359A**	13.3 ± 11.0	-6.5 ± 2.0	1.17 ± 1.23	8
**thTRESK WT/WT**	38.1 ± 1.9	262.2 ± 5.0	1.23 ± 0.05	11
**thTRESK WT/E755A**	40.1 ± 7.9	126.2 ± 9.5	1.16 ± 0.19	13
**thTRESK WT/Q765A**	24.8 ± 4.6	109.6 ± 7.8	1.47 ± 0.36	10

Supplementary Table 6 | Transition time of K4 evaluated by *in silico* electrophysiology. Occurrence time for 1st, 2nd, 3rd and 4th transition for each 100 ns simulation (Sim 1-3) is given. Further, mean values ± SEM are given for each channel variant.

	1st (S4→S3)	2nd (S3 →S2)	3rd (S2→S1)	4th (S1→ out)
**WT Sim 1**	2.7 ns	9.4 ns	35.1 ns	37.6 ns
**WT Sim 2**	1.3 ns	7.2 ns	15.4 ns	20.2 ns
**WT Sim 3**	0.6 ns	7.4 ns	40.6 ns	42.2 ns
**T322A Sim 1**	2.6 ns	14.6 ns	100.0 ns	100.0 ns
**T322A Sim 2**	1.2 ns	5.7 ns	100.0 ns	100.0 ns
**T322A Sim 3**	1.0 ns	12.8 ns	46.0 ns	52.2 ns
**WT Mean ± SEM**	1.5 ± 0.5 ns	8.0 ± 0.6 ns	30.4 ± 6.2 ns	33.3 ± 5.5 ns
**T322A Mean ± SEM**	1.6 ± 0.4 ns	11.0 ± 2.2 ns	82.0 ± 14.7 ns	84.1 ± 13.0 ns

Supplementary Table 7 | Loss of K1 / Rb1 and K4 / Rb4 in MD simulations without ESF. Occurrence time of K1 / Rb1 and K4 / Rb4 leaving the S1 or S4 binding site, respectively. If ions remain in the SF for the whole simulation time of the production run (150.0 ns) this is indicated by "-".

	K1 / Rb1	K4 / Rb4
**WT K^+ Sim 1**	-	10.0 ns
**WT K^+ Sim 2**	-	20.7 ns
**WT K^+ Sim 3**	-	-
**WT Rb^+ Sim 1**	1.0 ns	-
**WT Rb^+ Sim 2**	5.9 ns	-
**WT Rb^+ Sim 3**	1.3 ns	-
**T322A K^+ Sim 1**	-	-
**T322A K^+ Sim 2**	-	47.7 ns
**T322A K^+ Sim 3**	-	-
**T322A Rb^+ Sim 1**	4.7 ns	-
**T322A Rb^+ Sim 2**	4.0 ns	-
**T322A Rb^+ Sim 3**	5.5 ns	-

Supplementary Table 8 | TEVC recordings using 100 mM K^+ / Rb^+ solutions. Values for currents [µA] and ratios of currents elicited in 100 mM Rb^+ versus 100 mM K^+ in absence of cloxyquin (I_{Rb} / I_K) as well as ratios for current potentiation caused by 100 µM cloxyquin using different permeating ions (I_{clx} / I_{ctrl}) are given as mean ± SEM. Number of independent oocytes is given for measurements in absence (n_{ctrl} for currents and I_{Rb} / I_K) and presence (n_{clox} for I_{clx} / I_{ctrl}) of cloxyquin.

	**voltage**	**WT / K^+**	**WT / Rb^+**	**T322A / K^+**	**T322A / Rb^+**
**Current [µA]**	-120 mV	-3.48 ± 0.23	-6.58 ± 0.39	-0.80 ± 0.01	-1.32 ± 0.07
	+40 mV	2.41 ± 0.13	2.35 ± 0.12	0.29 ± 0.04	0.31 ± 0.03
**I_{Rb} / I_K**	-120 mV	1.91 ± 0.07		1.69 ± 0.09	
	+40 mV	0.98 ± 0.02		1.10 ± 0.06	
**n_{ctrl}**	-	22		20	
**I_{clx} / I_{ctrl}**	-120 mV	-2.60 ± 0.13	-2.03 ± 0.09	-3.02 ± 0.19	-4.02 ± 0.20
	+40 mV	2.94 ± 0.20	2.85 ± 0.26	3.16 ± 0.41	4.10 ± 0.53
**n_{clox}**	-	19	21	14	17

Supplementary Table 9 | Simulation setup parameters. All simulations were performed with 0.9 % NaCl salt concentration and membranes built up from 100 % phosphatidyl-ethanolamine (PEA).

**simulation type**	**protein type**	**run**	**simulation box (X x Y x Z)**	**atoms total**	**H_2O residues**	**Na^+ / Cl^- atoms**	**PEA residues**
equilibration	WT / T322A	1	123 x 157 x 123 Å	242,560	58,401	171 / 176	437
ion permeation (prod.)	WT	1	84 x 128 x 84 Å	91,947	20,556	62 / 63	163
		2	84 x 129 x 84 Å	92,464	20,520	62 / 63	168
		3	83 x 130 x 83 Å	92,286	20,544	62 / 63	166
ion permeation (prod.)	T322A	1	83 x 131 x 83 Å	91,917	20,548	63 / 64	163
		2	84 x 130 x 84 Å	92,387	20,497	62 / 63	168
		3	83 x 129 x 83 Å	91,860	20,488	62 / 63	164
ion occupancy for K^+ (prod.)	WT	1	83 x 133 x 83 Å	92,224	20,565	62 / 63	165
		2	83 x 131 x 83 Å	91,821	20,514	63 / 63	163
		3	82 x 134 x 82 Å	91,690	20,553	63 / 64	161
ion occupancy for K^+ (prod.)	T322A	1	83 x 130 x 83 Å	91,954	20,561	62 / 63	163
		2	82 x 132 x 82 Å	91,906	20,586	64 / 63	162
		3	83 x 130 x 83 Å	92,252	20,577	62 / 63	165
ion occupancy for Rb^+ (prod.)	WT	1	83 x 130 x 83 Å	92,135	20,452	63 / 62	167
		2	83 x 131 x 83 Å	91,973	20,564	63 / 64	163
		3	82 x 132 x 82 Å	91,703	20,599	63 / 64	160
ion occupancy for Rb^+ (prod.)	T322A	1	84 x 129 x 84 Å	92,477	20,402	63 / 62	171
		2	82 x 133 x 82 Å	91,752	20,577	63 / 63	161
		3	84 x 128 x 84 Å	92,380	20,453	62 / 63	169

SUPPLEMENTARY REFERENCES

1. Jumper, J. *et al.* Highly accurate protein structure prediction with AlphaFold. *Nature* **596**, 583–589 (2021).

2. Varadi, M. *et al.* AlphaFold Protein Structure Database: massively expanding the structural coverage of protein-sequence space with high-accuracy models. *Nucleic Acids Res* **50**, D439–D444 (2022).

3. Wicha, S. G., Chen, C., Clewe, O. & Simonsson, U. S. H. A general pharmacodynamic interaction model identifies perpetrators and victims in drug interactions. *Nat Commun* **8**, (2017).
