# Supplementary material for: Cloxyquin activates hTRESK by allosteric modulation of the selectivity filter
Source: Commun Biol. 2023 Jul 18;6:745. doi: 10.1038/s42003-023-05114-4 (PMC10354012; doi:10.1038/s42003-023-05114-4)
Supplement: Supplementary file 2 — Description of Additional Supplementary Files [file 42003_2023_5114_MOESM2_ESM.pdf]

Description of Additional Supplementary Files

File name: Supplementary Data 1

Description: Source data for the main figures

File name: Supplementary Data 2

Description: First and Final snapshots of simulations.
